# p16^INK4a^ Plays Critical Role in Exacerbating Inflammaging in High Fat Diet Induced Skin

**DOI:** 10.1155/2022/3415528

**Published:** 2022-11-21

**Authors:** Yan Liang, Tianya Gu, Su Peng, Yi Lin, JiaBao Liu, Xiaoyan Wang, Xin Huang, Xiaodong Zhang, Jun Zhu, Lin Zhao, Changyan Fan, Guangyan Wang, Xin Gu, JinDe Lin

**Affiliations:** ^1^Department of Plastic Surgery, The Affiliated Friendship Plastic Surgery Hospital of Nanjing Medical University, Nanjing, Jiangsu 210029, China; ^2^Department of Cardiology, The First Affiliated Hospital of Nanjing Medical University, Nanjing, Jiangsu 210029, China; ^3^Department of Cardiology, The Affiliated Hospital of Jiangnan University, Wuxi, Jiangsu 214062, China

## Abstract

**Background:**

Long term high fat diets (HFD) promote skin aging pathogenesis, but detailed mechanisms remain unclear especially for inflammaging, which has recently emerged as a pathway correlating aging and age-related disease with inflammation. p16^INK4a^ (hereafter termed p16) inhibits the cell cycle, with p16 deletion significantly inhibiting inflammaging. We observed that HFD-induced p16 overexpression in the skin. Therefore, we investigated if p16 exacerbated inflammaging in HFD-induced skin and also if p16 deletion exerted protective effects against this process.

**Methods:**

Eight-week-old double knockout (KO) ApoE^−/−^p16^−/−^ mice and ApoE^−/−^ littermates were fed HFD for 12 weeks and their skin phenotypes were analyzed. We measured skin fibrosis, senescence-associated secretory phenotype (SASP) levels, and integrin-inflammasome pathway activation using histopathological, RNA-sequencing (RNA-seq), bioinformatics analysis, and molecular techniques.

**Results:**

We found that HFD contributed to inflammaging in the skin by activating the NLRP3 inflammasome pathway, increasing inflammatory infiltration, and promoting apoptosis by balancing expression between proapoptotic and antiapoptotic molecules. p16 knockout, when compared with the ApoE^−/−^ phenotype, inhibited skin fibrosis by ameliorating inflammatory infiltration and proinflammatory factor expression (Interleukin-1*β* (IL-1*β*), Interleukin-6 (IL-6), and tumor necrosis factor-*α* (TNF-*α*)), and also alleviated inflammaging skin progress induced by HFD in the ApoE^−/−^ mouse model. RNA-seq showed that p16 KO mice inhibited both integrin-inflammasome and NF-*κ*B proinflammatory pathway activation.

**Conclusions:**

p16 deletion or p16 positive cell clearance could be a novel strategy preventing long term HFD-induced skin aging.

## 1. Introduction

In society in recent years, obesity/overweight prevalence has increased significantly [1]. Accordingly, the incidence of many obesity-related diseases such as diabetes and non-alcoholic fatty liver disease has increased [2]. Previous studies have showen that dermatitis development was closely related to systemic diseases, including type 2 diabetes [3], hyperlipidemia [4], and coronary artery disease. Bonomini et al. reported that ApoE knockout (ApoE^−/−^) mice exhibited several aging phenotypes, such as skin thinning, epidermal thickening, hair follicle loss, and premature graying, with hair regeneration disorders [5]. After feeding a high-fat diet (HFD) to ApoE^−/−^ mice, their skin showed a thinning of non-xanthomatous lesions and skin aging phenotypes such as collagen fiber disorder [6]. However, the precise mechanisms of how HFD exacerbated dermatitis are unclear.

With increasing age, the skin becomes thinner, paler, and wrinkled due to endogenous and exogenous factors [7]. Although the underlying mechanisms of aging skin pathogenesis are not completely clear, multiple pathway involvement has been proposed. Inflammaging was firstly named by C Franceschi [8]. The concept was originally intended to describe the chronic, low-level inflammatory state that accompanies the aging processes in immune cell senescence (immunosenescence) [8]. Inflammaging has recently emerged as a pathway that correlates aging and age-related disease with inflammation, and is characterized by increased circulating proinflammatory cytokines and a shift toward cell senescence, changes believed to drive many age-associated conditions, including dementia, arthritis, type 2 diabetes,and skin aging [9, 10]. Laurentius *at el.* reported that HFD diet aggravated inflammatory and fibrotic microenvironments in aging rat kidneys [11], and HFD altered gut microbiota to promote intestinal inflammation [12].

p16^INK4a^ (p16) is encoded by *CDKN2A* on chromosome 9 and binds with cyclin dependent kinases 4 and 6 to decrease Rb phosphorylation and cause cell cycle arrest and cell senescence [13]. p16 deletion dramatically prevents cell senescence and ameliorates renal senescence associated tubulointerstitial fibrosis in a stress-induced premature senescence mouse model [14]. Clearance of p16 positive senescent cells in mice alleviates the detrimental features of cardiac aging, including myocardial hypertrophy and fibrosis [15]. We previously showed that p16 promoted acute tubular necrosis development by increasing inflammatory infiltration, proinflammatory factor expression, and regulating reactive oxygen species levels via PGAM5-KEAP1 signaling [16]. However, it is unclear if p16 accumulation in aging skin is involved in HFD-induced skin changes.

NLRP3 inflammasome is a protein complex that recognize a diverse array of extracellular and intracellular signals, including damage-associated and pathogen-associated molecular patterns (DAMPs and PAMPs, respectively). NLRP3 inflammasome pathway activation induces proinflammatory cytokines such as interleukin-1*β* (IL-1*β*) and IL-18 to generate a proinflammatory microenvironment and inflammaging [17]. However, it is unclear if p16 activates the NLRP3 inflammsome pathway and aggravates inflammaging.In our study, ApoE^−/−^ and ApoE^−/−^p16^−/−^ mice were used to establish hyperlipidemia model. Senescent cells and senescence-associated secretory phenotypes (SASP) in skin tissue were investigated from an inflammaging perspective. Additionally, senescence related protein expression and SASP levels in fibroblasts induced by oleic acid and palmitic acid were studied *in vitro* and *in vivo*. Also, transcriptomics analyses identified mechanisms potentially involving HFD-induced p16 over-expression could activate integrin-inflammasome pathway, and other NF-*κ*B signaling involved in inflammatory skin aging regulation. We explored how chronic inflammation, caused by HFD, accelerated skin aging, and we investigated molecular mechanisms underlying this process. Our data could provide new treatments for delaying skin aging and other related diseases.

## 2. Materials and Methods

### 2.1. Study Animals

*p16* heterozygote male and female mice in the FVB N2 background were mated to generate *p16KO* mice (*p16*^−/−^ with exon 1*α* deleted) and wild-type littermates. Mice were genotyped as previously described [17]. Eight-week-old male ApoE knockout (ApoE^−/−^) mice were purchased from the Model Animal Research Center, Nanjing University and crossed with *p16*^−/−^ mice to generate ApoE^−/−^*p16*^−/−^ animals. Eight-week-old male ApoE^−/−^ and ApoE^−/−^*p16*^−/−^ mice were used in this study. All experiments were conducted using at least six mice. All studies were conducted according to Experimental Animal Research Institute of Nanjing Medical University guidelines. The study was approved by the Nanjing Animal Experimental Ethics Committee (Permit Number; IACUC-1808012).

### 2.2. The HFD Mouse Model

Eight-week-old ApoE^−/−^ and ApoE^−/−^p16^−/−^ mice were fed HFD diet (D12108C, SYSE Bio-Tec, Changzhou, China) for 12 weeks. Animals were weighed weekly. The HFD contained 60% calories. The normal diet (ND) contained 20% calories. After 12 weeks, skin tissues were obtained for analysis. Composition of the HFD diet and normal diet were shown in Table S2.

### 2.3. Cell Culture and Oil Red Staining

Primary human dermal fibroblasts (HDFs) were cultured as previously described [18, 19]. Fifteen foreskin samples were collected from healthy males. None of the donors had any medical conditions or were under medication. Briefly, HDFs were isolated from the tissue specimens by enzymatic digestion using collagenase (Roche). Cells were cultured in Dulbecco's Modified Eagle's Media-high glucose (DMEM; Sigma-Aldrich, St. Louis, MO, USA) supplemented with 10% fetal bovine serum and 1% penicillin/streptomycin at 37°C in a 5% CO_2_ humidified incubator. To induce steatosis, we used human dermal fibroblasts (HDFs) using sodium palmitate (10 mmol/L, Sigma-Aldrich) and sodium oleate (10 mmol/L, Sigma-Aldrich) for 24 h. For Oil Red staining, Lipid Stain were purchased from Abcam (ab150678) and used the following manufacturer's instructions.

### 2.4. Transfection of p16-Overexpression Adenovirus

*p16 (human)* overexpression adenovirus carrying the Flag-tag was designed and synthesized by Genechem Co., Ltd., Shanghai, China. The adenovirus was transfected into HDF cells and incubated with antibiotic-free complete medium at 60%–70% confluence for 6 h. After this, cells were cultured in Dulbecco's Modified Eagle Media-high glucose supplemented with 10% fetal bovine serum. After 48 h, HDFs were induced using sodium palmitate and sodium oleate (10 mmol/L, Sigma-Aldrich) for further analysis.

### 2.5. Skin Processing

Mice were anesthetized with 3% sodium pentobarbital (40 mg/kg). Skin tissue was excised and washed in 100 ml phosphate buffered saline (composition of the saline solution were shown in Table S3). Samples were cut into pieces and fixed in periodate-lysine-paraformaldehyde (for histochemistry and immunohistochemistry (IHC) overnight at 4°C. For hematoxylin & eosin (H&E) or immunohistochemical staining, sections were dehydrated in a gradient ethanol series, embedded in paraffin, and 5 *μ*m sections were cut using a microtome (Leica Microsystems Nussloch GmbH, Nucloch, Germany) as previously described, siRNA transfection and virus infection.

ITGAL and ITGAM siRNA directed against human ITGAL or ITGAM, and negative control siRNA were synthesized by Guangzhou RiboBio Co., Ltd., China. HDFs cells were transfected with 50 nM of control or ITGAL or ITGAM siRNA using lipofectamine 2000 (Thermo Fisher Scientific, USA) and Opti-MEM I Reduced Serum Medium (Gibco, USA) following the standard procedure. After siRNA treatment, the cells were incubated for 6 h, and then the medium was replaced with fresh medium. Real-time qPCR and Western Blot were used to confirm efficiency of transfection. The primer sequence of NC and ITGAL or ITGAM siRNA were shown in Table S4.

### 2.6. Western Blotting

Skin tissues were homogenized in radioimmunoprecipitation assay buffer (New Cell & Molecular Biotech Co., Ltd.) containing protease inhibitors. Total protein was measured in supernatants (Bicinchoninic acid assay kit, Thermo Fisher Scientific Co.). Western blotting was conducted as previously described [13, 20]. In this study, 30 *μ*g protein was used for analysis. Primary antibodies against p16 (ab211542), p16 (ab152099), p19 (ab80), tumor necrosis factor-*α* (TNF-*α*) (ab183218), IL-1*β* (ab9722), NLRP3 (ab263899), ApoE (ab183597), NLRC4 (ab201792), and ITGAM (ab133357) were purchased from Abcam (Cambridge, MA, USA). Caspase-1 (#83383), p53 (#2524), p65 (#8242), p-p65 (Ser536) (#3033), Bax (#2772), and Cleaved caspase 3 (#9664) were purchased from Cell Signaling Technology (Beverly, MA, USA). GAPDH (60004-1-Ig), *β*-galactosidase (15518-1-AP), Bcl-2 (12789-1-AP), Collagen I (14695-1-AP), Collagen III (22734-1-AP), and *α*-SMA (14395-1-AP) were purchased from Proteintech (Rosemont, IL, USA). ASC (GTX55818) and IL-6 (GTX110527) were purchased from GeneTex (Centennial, CO, USA). Horse radish peroxidase (HRP)-conjugated, Affinipure Goat Anti-Rabbit IgG (H + L), and HRP-conjugated Affinipure Goat Anti-Mouse IgG (H + L) were purchased from Proteintech (USA). Protein signals were visualized using an enhanced chemiluminescent solution (Millipore Sigma, WBKL S0500) and detected using an UVitec Alliance mini-chemiluminescence device (UVitec, UK). Western blot band intensity was quantified using ImageJ software.

### 2.7. Histology

Immunohistochemistry (IHC) staining was performed according to a previous method [13, 20]. Serial paraffin sections were subjected to antigen retrieval, incubation in antigen retrieval solution for 20 min, inactivation with endogenous peroxidase (3% H_2_O_2_), and blocked in goat serum for 1 h. Sections were then incubated with primary antibodies against CD3 (SC-20047, Santa Cruz Biotechnology Inc., Dallas, TX, USA), F4/80 (SC-377009, Santa Cruz Biotechnology Inc.), IL-6 (GeneTex, Santa Cruz Biotechnology Inc.), IL-1*β* (ab9722, Abcam), TNF-*α* (ab183218, Abcam), p-p65 (Ser536) (#3033, Cell Signaling Technology), p16 (ab211542, Abcam), NLRC4 (ab201792, Abcam), ITGAM (ab133357, Abcam), p19 (ab80, Abcam), Collagen I (14695-1-AP, Proteintech), Collagen III (22734-1-AP, Proteintech), *α*-SMA (14395-1-AP, Proteintech), and *β*-galactosidase (15518-1-AP, Proteintech). After washing, sections were incubated with a secondary antibody for 1 h, and processed using the SABC-POD kit (SA2001, Boster, China). Then, sections were counterstained with hematoxylin and fixed with biomount medium. Hematoxylin and Shandon Instant Eosin (Solarbio Co., Ltd.) were used to determine cell infiltration. Masson's trichrome staining (Sigma-Aldrich®) was used to assess collagen deposition.

### 2.8. Quantitative Real-Time Polymerase Chain Reaction (qRT-PCR)

RNA was isolated from skin tissue using Trizol reagent (Thermo Fisher Scientific) as previously described [20]. Reverse transcription reactions were performed using the HiScript III 1^st^ strand cDNA synthesis kit according to manufacturer's instructions (Vazyme). Next, qRT-PCR was conducted using a FastStart™ SYBR green mix kit (Sigma-Aldrich), and reactions is performed on an Applied Biosystems 7300 RT PCR system according to manufacturer's protocols. GAPDH (housekeeping gene) (CT_control_) and target gene expression levels (CT_target gene_) were determined. Normalized target gene expression (*Δ*CT_target gene_) was calculated (CT_target gene_ − CT_control_). Expression fold changes in target genes were calculated (2^–ΔCT^ target gene) in triplicate samples. Primer sequences are shown in Table S1.

### 2.9. RNA-Seq and Bioinformatics Analysis

RNA was isolated from HFD-induced skin from ApoE^−/−^p16^−/−^ knockout and ApoE^−/−^ mice. RNA-seq were measured as previously described [20]. cDNA sequencing libraries were prepared using the TruePrep DNA Library Prep Kit V2 for the Illumina platform and subjected to 2 × 150 paired-end sequencing. To identify differentially expressed genes, fold expression changes were calculated for each gene by dividing the average fragments per kilobase of transcript per million mapped reads for the case by the average fragments per kilobase of transcript per million mapped reads for the control. Gene Ontology (GO) and Kyoto Encyclopedia of Genes and Genomes (KEGG) terms were analyzed in Database for Annotation, Visualization, and Integrated Discovery (DAVID). STRING analysis was used to show protein interaction networks and PANTHER analysis was used to show molecular functions and gene pathways.

### 2.10. Statistical Analysis

Measurement data were described as the mean ± standard error of the mean fold-change over controls and analyzed using Student's *t*-test and one-way analysis of variance to compare differences among groups. Qualitative data were described as percentages and analyzed using chi-square tests. All analyses were performed using SPSS (Version 19.0; SPSS Inc., Chicago, IL, USA) or GraphPad Prism software (Version 6.02) as previously described. *p* < 0.05 was considered statistically significant.

## 3. Results

### 3.1. HFD Induces p16 Overexpression and Accumulation in Skin Senescent Cells

Previous studies reported close links between HFD and skin disease. Herbert et al. observed that HFDs exacerbated early psoriatic skin inflammation by promoting proinflammatory stimulation of free fatty acids [21]. Additionally, other studies identified proinflammatory effects from HFDs in multiple dermatitis mouse models. Recently, the protective effects of senescent cell clearance was observed during hair loss in aging mice. Senescent cells contribute to fibrotic and inflammatory pathogenesis in multiple tissues [22]. The clearance of senescent cells could alleviate multiorgan liver, kidney, and lung fibrosis [23, 24]. Unfortunately, it is unclear if skin phenotypes induced by HFDs exhibit senescent cell accumulation.

**Figure 1:**
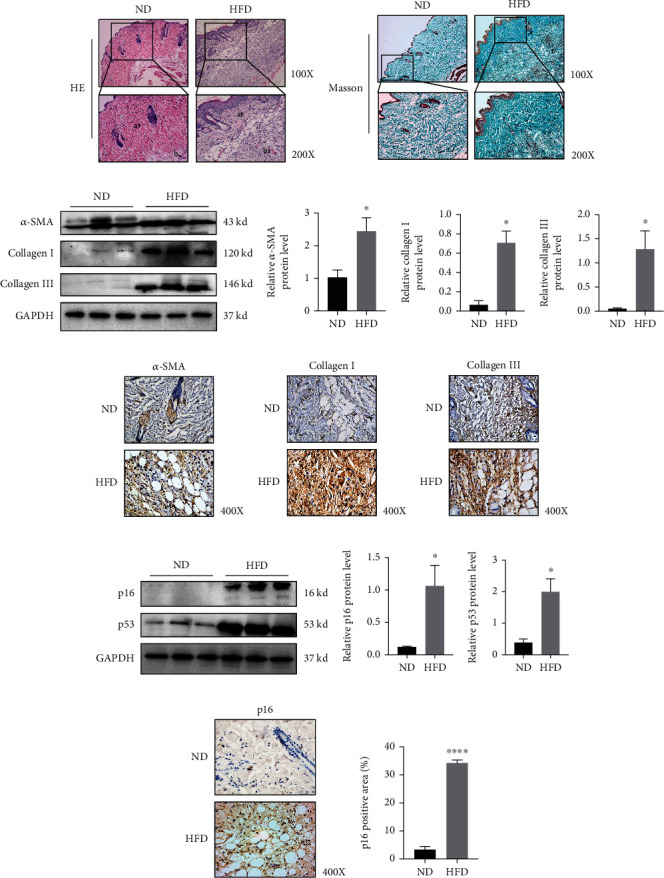
High fat diet induces p16 overexpression and accumulation in skin senescent cells. Eight-week-old ApoE^−/−^ mice were fed with normal diet (ND) and High fat diet (HFD) for 3 months and obtained skin tissues for further analysis. (a) H&E staining showing inflammatory infiltration of skin from 20-week-old ApoE^−/−^ mice induced by ND and HFD (*n* = 3); (b) representative images of Masson staining to assess skin collagen deposition from 20-week-old ApoE^−/−^ mice induced by ND and HFD (*n* = 3); (c–f) expression levels and statistical figures of *α*-SMA, Collagen I, and Collagen III in skin tissues from 20-week-old ApoE^−/−^ mice induced by ND and HFD were detected by western blotting (*n* = 3); (g–i) expression levels of *α*-SMA, Collagen I, Collagen III, and p16 in skin tissues from 20-week-old ApoE^−/−^ mice induced by ND and HFD by immunohistochemical staining (*n* = 3); (j–l) Expression levels and statistical figures of p16 and p53 in skin tissues from 20-week-old ApoE^−/−^ mice induced by ND and HFD by western blotting (*n* = 3); (m, n) expression levels and statistical figures of p16 in skin tissues from 20-week-old ApoE^−/−^ mice induced by ND and HFD by immunohistochemical staining (*n* = 3); values are mean ± SEM, ^∗^*p* < 0.05, ^∗∗∗^*p* < 0.001, ^∗∗∗∗^*p* < 0.0001 compared with ND mice.

To clarify this issue, we fed ApoE knockout (*ApoE*^−/−^) mice a ND (20% calories) and HFD (60% calories) for three months. Tissues were stained with H&E and Masson stain to identify histopathological changes in the skin of *ApoE*^−/−^ mice in the HFD group relative to the ND group. H&E staining showed that the epidermis of HFD animals was significantly thickened when compared with ND animals (Figure 1(a)). Also, HFD skin showed dermal collagen with loose and disordered tissue, and more fat cavitations relative to ND mice (Figure 1(a)). We also observed fewer fibroblasts (Figure 1(a), arrow a) and more infiltrating inflammatory cells when compared with the ND mice (Figure 1(a), arrow b), suggesting skin inflammation in HFD animals. Masson trichrome staining showed that while collagen fiber expression levels increased, collagen fibers in the skin were looser and more disordered in HFD-fed group, thereby decreasing skin elasticity and impairing skin barrier function (Figure 1(b)). Thus, HFD may have accelerated skin fibrosis. To clarify this, western blotting of the profibrotic proteins, *α*-SMA, Collagen I, and Collagen III showed that HFD induced their overexpression (Figures 1(c)–1(f)). While collagens are important structural scaffold skin proteins, they require an ordered and structured system. The expression levels of these profibrofic proteins (*α*-SMA, Collagen I, and Collagen III) in skin were also detected by IHC stain (Figures 1(g)–1(i) and Figure S1(a)), with results similar to immunoblotting data.

We also examined the differential expression profiles of the senescence-related proteins, p16 and p53 by western blotting. When compared with the ND group, HFD induced p16 overexpression and increased p53 expression levels, suggesting senescent cell accumulation in the skin (Figures 1(j)–1(l)). To verify this, we investigated p16, p19, and *β*-galactosidase expression by IHC and observed p16, p19 overexpression in the derma and elevated *β*-galactosidase positive (*β*-gal^+^) cells in HFD when compared with ND samples (Figures 1(m)–1(n) and Figures S1(b) and S1(c)). Additionally, in the HFD group, we observed increased p19 expression (Figures S1(c) and S1(d)) which is a downstream p16 protein, and increased expression of another senescent cell marker, p21 by western blotting (Figure S1(d)). Therefore, HFD induced p16 overexpression and accumulation of senescent cells in the derma.

### 3.2. HFD Induces Inflammaging in the Skin of ApoE^−/−^ Mice

**Figure 2:**
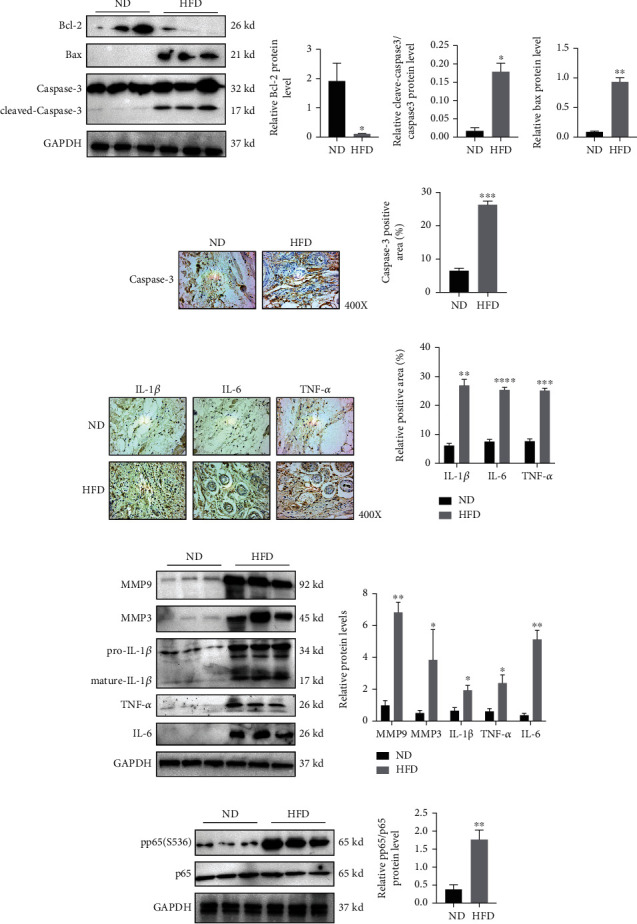
High fat diet induces inflammaging in skin of ApoE^−/−^ mice. Eight-week-old ApoE^−/−^ mice were fed with normal diet (ND) and High fat diet (HFD) for 3 months and obtained skin tissues for further analysis. (a–d) Expression levels and statistical figures of Bcl-2, Bax, and cleaved-caspase-3 in skin tissues from 20-week-old ApoE^−/−^ mice induced by ND and HFD by western blotting (*n* = 3); (e, f) representative images and statistical analysis of Caspase-3 in skin by immunohistochemical staining (*n* = 3); (g, h) representative images and statistical analysis of IL-1*β*, IL-6, and TNF-*α* in skin by immunohistochemical staining (*n* = 3); (i, j) expression levels and statistical figures of MMP9, MMP3, pro-IL-1*β*, mature-IL-1*β*, TNF-*α*, and IL-6 in skin tissues from 20-week-old ApoE^−/−^ mice induced by ND and HFD by western blotting (*n* = 3); (k, l) expression levels and statistical figures of pp65(S536) and p65 in skin tissues from 20-week-old ApoE^−/−^ mice induced by ND and HFD by western blotting (*n* = 3); values are mean ± SEM, ^∗^p < 0.05, ^∗∗^*p* < 0.01, ^∗∗∗^*p* < 0.001, ^∗∗∗∗^*p* < 0.0001 compared with ND group mice.

As HFD induced p16 overexpression and accumulation in skin senescent cells, we hypothesized if the skin of ApoE^−/−^ mice fed with HFD would display characteristic inflammaging phenotypes. By western blotting, we identified varying levels of different proapoptosis or antiapoptosis associated proteins. When compared with the ND group, HFD-induced mice expressed higher Bax and cleaved-caspase-3 levels, but less Bcl2 levels, suggesting the HFD increased apoptosis rates in the skin (Figures 2(a)–2(d)). Also, we found that HFD increased caspase-3 levels in skin by IHC staining (Figures 2(e) and 2(f)).

Inflammaging represents low-grade, chronic, sterile systemic inflammation during aging, and is a highly significant risk factor for morbidity and mortality in elderly individuals. To investigate inflammatory infiltration of skin tissue in ApoE^−/−^ mice after HFD, we performed IHC to identify CD3 and F4/80 positive cell percentages in these animals (Figures S2(a) and S2(b)). Percentages in the HFD-fed group were significantly increased when compared with ND groups. Therefore, HFD promoted inflammatory infiltration in the skin of ApoE^−/−^ mice.

We next hypothesized if HFD may increase inflammatory factor production and inflammaging levels in the skin. To address this, IHC was used to investigate IL-1*β*, IL-6, and TNF-*α* expression levels in the skin of ApoE^−/−^ mice (Figure 2(g)). IL-1*β*-positive, IL-6-positive, and TNF-*α*-positive cell or region percentages were significantly increased in HFD mice when compared with ND group mice (Figure 2(h)). We also confirmed these findings by western blotting (Figures 2(i)–2(j)). We also observed that MMP9 and MMP3 expression levels were increased after HFD (Figures 2(i)–2(j)). Combined, these findings suggested that HFD more likely increased leukocyte recruitment to the skin of HFD-fed mice via proinflammatory cytokine production.

Previous studies showed that NF-*κ*B (p65/RelA) functions as a master regulator of SASP, controlling both cell-autonomous and noncell-autonomous aspects of the senescence program. Recently, NF-*κ*B activation was identified as an inflammaging marker phenotype [25]. From our results, when compared with the ND group, phospho-NF-*κ*B p65 levels at the 536 serine site were increased significantly in the HFD group (Figures 2(k) and 2(l) and Figure S2(c)). Therefore, HFD aggravated inflammaging phenotypes in the skin of ApoE^−/−^ mice.

### 3.3. p16 Overexpression Aggravates Inflammaging Phenotypes in HDFs

**Figure 3:**
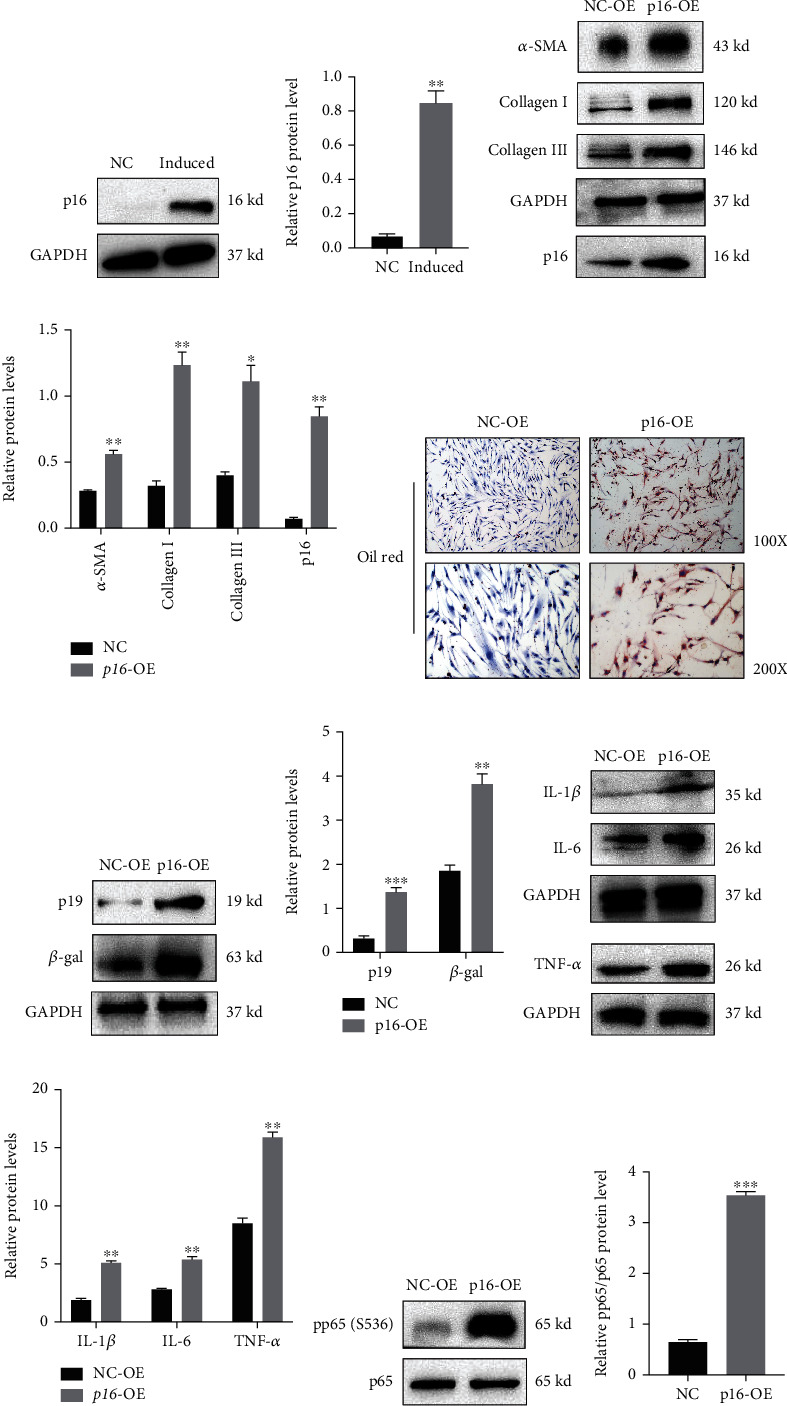
Steatosis induced or p16 overexpression aggravates inflammaging phenotypes in human dermal fibroblasts. Human dermal fibroblasts (HDFs) were induced steatosis for 24 h under medium containing sodium palmitate (10 mmol/L) and sodium oleate (10 mmol/L); HDF cells were transfected with NC and p16 overexpression adenovirus for further analysis. (a, b) Expression levels and statistical figures of p16 in skin fibroblasts and induced steatosis of skin fibroblasts by western blotting (*n* = 3); (c, d) expression levels and statistical figures of *α*-SMA, Collagen I, Collagen III, and p16 in skin fibroblasts transfected with NC and p16 overexpression adenovirus by western blotting; (e) representative images of Oil Red staining in skin fibroblasts transfected with NC and p16 overexpression adenovirus (*n* = 3); (f, g) expression levels and statistical figures of p19, *β*-galactosidase in skin fibroblasts transfected with NC, and p16 overexpression adenovirus; (h, i) expression levels and statistical figure of IL-1*β*, IL-6, and TNF-*α* in skin fibroblasts transfected with NC and p16 overexpression adenovirus by western blotting; (j, k) expression levels and statistical figures of p65, pp65(S536) in skin fibroblasts transfected with NC, and p16 overexpression adenovirus by western blotting (*n* = 3); values are mean ± SEM, ^∗^*p* < 0.05, ^∗∗^*p* < 0.01, ^∗∗∗^*p* < 0.001 compared with NC group.

As HFD induced *in vitro* p16 overexpression, we induced steatosis in HDFs using sodium palmitate (10 mmol/L) and sodium oleate (10 mmol/L) for 24 h to examine if p16 induced fibroblast senescence and SASP secretion. We observed increased p16 expression in fibroblasts after inducing steatosis (Figures 3(a) and 3(b)). Next, HDFs were transfected with NC and p16 overexpression adenoviruses to further clarify the profibrosis effects of p16 overexpression, we examined *α*-SMA, Collagen I, and Collagen III levels in HDFs and observed that p16 induced upregulation of these proteins, thereby inducing profibrotic effects in HDFs (Figures 3(c) and 3(d)). Also, we found that p16 overexpression increased steatosis levels in HDFs by Oil Red stain (Figure 3(e)). Also, western blotting was performed to assess expression of the senescence related proteins, p19 and *β*-galactosidase. Our results showed that p16 overexpression in HDFs induced overexpression of p19 and *β*-galactosidase (Figures 3(f) and 3(g)). Additionally, we investigated SASP cytokine expression in HDFs and observed that p16 overexpression increased IL-1*β*, IL-6, and TNF-*α* levels (Figures 3(h)–3(i)). Also, p16 overexpression increased pp65 (S536) levels in HDFs (Figures 3(j)–3(k)). Thus, p16 overexpression in HDFs aggravated the inflammaging phenotype. Furthermore, we had detected expression level of ApoE in HDFs and found that after inducing steatosis via sodium palmitate and sodium oleate, ApoE expression in HDFs. In addition, we found that p16 overexpression could increase expression level of ApoE in HDFs after inducing steatosis (Figures S3(a) and S3(b)). These interesting results indicated that p16 and ApoE might form positive regulatory network in skin aging induced by high fat diet.

### 3.4. Transcriptomics Identifies the Inhibitory Effects of Proinflammatory Responses in the Skin of p16 Knockout Mice

**Figure 4:**
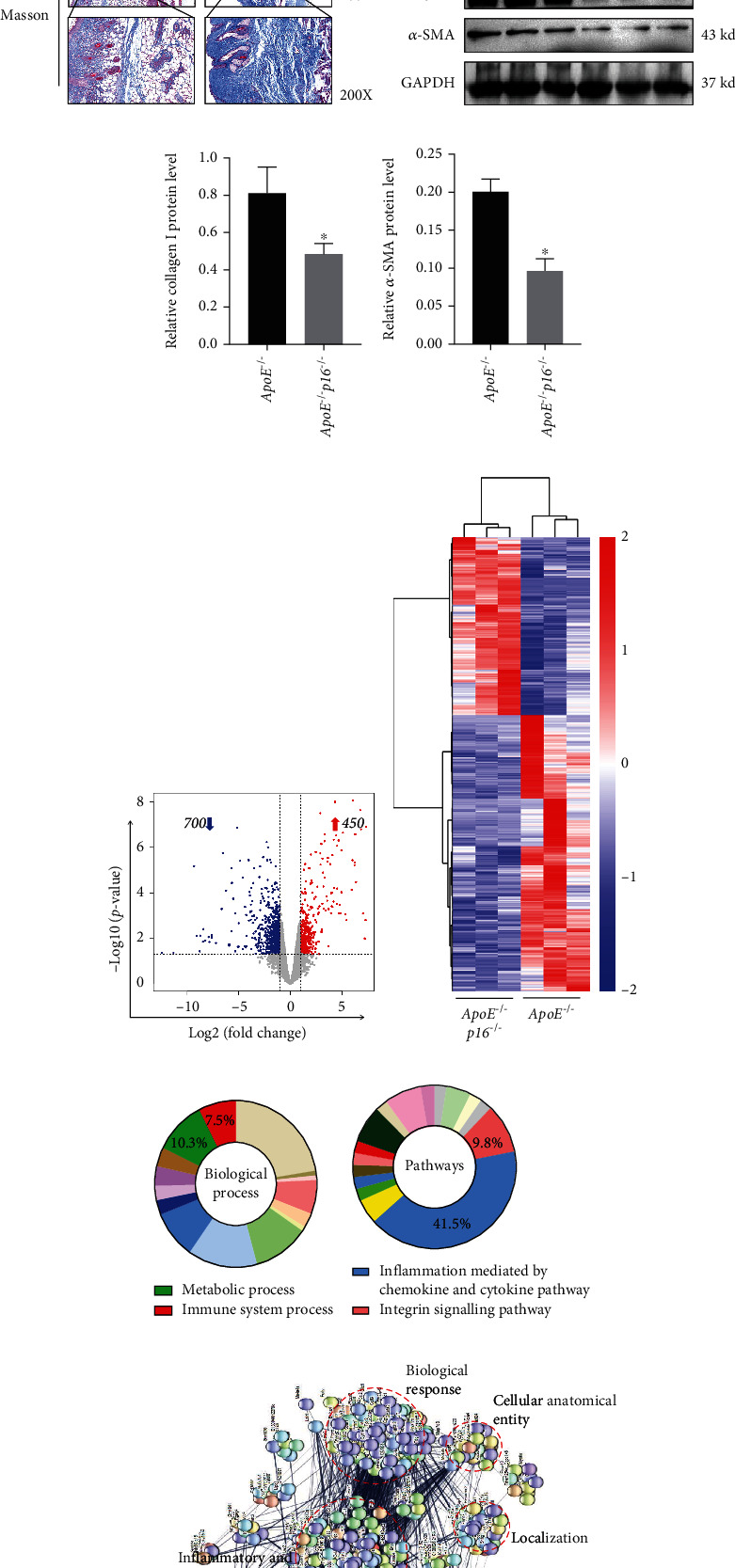
Transcriptomics identify the inhibitory effects of proinflammatory responses in the skin of p16 knockout mice. Eight-week-old ApoE^−/−^ and ApoE^−/−^p16^−/−^ were fed with HFD for 12 weeks and obtained skin tissues for further analysis. (a) Pattern diagram of mice experiment; (b) H&E staining of skin from 20-week-old ApoE^−/−^ and ApoE^−/−^p16^−/−^ mice induced by HFD (*n* = 3); (c) representative images of Masson staining to assess skin collagen deposition from 20-week-old ApoE^−/−^ and ApoE^−/−^p16^−/−^ mice induced by HFD (*n* = 3); (d–f) expression levels and statistical figures of Collagen I and *α*-SMA in skin tissues from 20-week-old ApoE^−/−^ and ApoE^−/−^p16^−/−^ mice induced by HFD by western blotting (*n* = 3); (g) RNA-seq analysis on HFD-induced skin (*n* = 3), identified 1150 statistically significant (*p* < 0.05) differentially expressed genes which were presented as a volcano plot: fold change from ApoE^−/−^ vs. ApoE^−/−^p16^−/−^; (h) differentially expressed genes by RNA-seq were shown as Heatmap; (i, j) functional profiling shown Biological Process of all differentially expressed genes identified by RNA-seq using Panther Classification System; (k) representative protein–protein interaction (PPI) network built with all downregulation significantly altered genes using STRING. Further functional analysis was performed with DAVID and significantly enriched categories are highlighted; values are mean ± SEM, ^∗^*p* < 0.05, compared with ApoE^−/−^ mice.

As HFD induced p16 overexpression, we hypothesized that *p16* knockout (KO) could alleviate the inflammaging phenotype. To investigate this, ApoE^−/−^ mice were crossed with p16^−/−^ mice to generate ApoE^−/−^p16^−/−^ animals which were then subjected to HFD for 12 weeks (Figure 4(a)). H&E and Masson staining showed that ApoE^−/−^p16^−/−^ mice were protected from derma fibrosis after being fed with HFD; they displayed tightly arranged skin collagen fibers and showed fewer infiltrating inflammatory cells when compared with ApoE^−/−^ mice (Figures 4(b) and 4(c)). Accordingly, we observed statistically significant differences between groups with respect to Collagen I and *α*-SMA expression (Figures 4(d)–4(f)). Therefore, skin fibrosis in HFD-induced ApoE^−/−^ mice was alleviated by *p16* ablation.

To explore mechanisms underpinning the p16 regulation of HFD-induced skin fibrosis, we processed skin tissue from ApoE^−/−^ and ApoE^−/−^*p16*^−/−^ mice using RNA-seq and found that the expression levels of 1150 genes were altered post-p16 deletion. Among genes, 450 were upregulated and 700 downregulated (Figures 4(g) and 4(h)). Biological processing terms from PANTHER analysis showed that metabolic and immune system processes were enriched for all downregulated genes (Figure 4(i)). Pathway terms from PANTHER analysis showed that inflammation associated and integrin pathways were also enriched (Figure 4(j)). Bioinformatics analysis also indicated that p16 deletion inhibited inflammation responses and the integrin pathway in the skin of ApoE^−/−^ mice (Figure 4(j)). Furthermore, the construction of a protein interaction network in STRING showed that p16 knockout mainly affected eight biological processes: inflammatory and immune responses, biological responses, response to lipids, intracellular signaling transduction, cellular anatomical entity, cell adhesion, and oxidation reduction processes and locations (Figure 4(k)). These data suggested that p16 deficiency inhibited proinflammatory processes in the skin of HFD-induced ApoE^−/−^ mice.

### 3.5. HFD or p16 Overexpression Activates the NLRP3 Inflammasome Pathway

**Figure 5:**
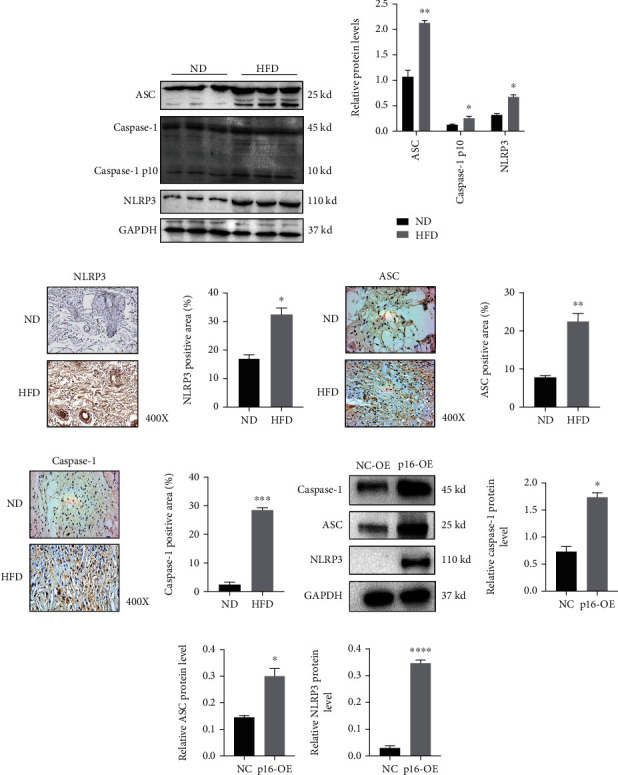
HFD or p16 overexpression activates the NLRP3 inflammasome pathway. Eight-week-old ApoE^−/−^ mice were fed with normal diet (ND) and high fat diet (HFD) for 3 months and obtained skin tissues for further analysis. (a, b) Expression levels and statistical figures of ASC, Caspase-1, Caspase-1 p10, and NLRP3 in skin tissues from 20-week-old ApoE^−/−^ mice induced by ND and HFD by western blotting (*n* = 3); (c–h) expression levels and statistical analysis of NLRP3, ASC, and Caspase-1 in skin tissues from 20-week-old ApoE^−/−^ mice induced by ND and HFD by immunohistochemical staining (*n* = 3); (i–l) human dermal fibroblasts (HDF) were induced steatosis for 24 h under medium containing sodium palmitate (10 mmol/L) and sodium oleate (10 mmol/L); HDF cells were transfected with NC and p16 overexpression adenovirus for further analysis. Expression levels and statistical figures of Caspase-1, ASC, and NLRP3 in skin fibroblasts transfected with NC and p16 overexpression adenovirus by western blotting (*n* = 3); values are mean ± SEM, ^∗^*p* < 0.05, ^∗∗^*p* < 0.01, ^∗∗∗^*p* < 0.001, ^∗∗∗∗^*p* < 0.0001 compared with ND or NC group.

NLRP3 has gained considerable translational attention in the inflammation research field. After activation, NLRP3 undergoes conformational alterations and interacts with the adaptor protein, ASC, which then bridges NRLP3 to procaspase-1 via its caspase activation and recruitment domain [26]. This activates caspase-1 to induce the maturation and secretion of various proinflammatory cytokines, including pro-IL-1*β* and pro-IL-18 [17]. Studies have shown that NLRP3 may initiate NF-*κ*B activation to in pathogen-induced or sterile inflammation. Also, as previous studies showed that HFDs induced the inflammasome-ASC complex via lipid metabolism regulation and oxidative stress [27, 28], we examined if HFD affected NLRP3 inflammasome pathway activation in the skin. Western blotting showed that NLRP3, ASC, Caspase-1, and Caspase-1 p10 expression levels were increased after HFD administration (Figures 5(a) and 5(b)). Additionally, when compared with the ND group, the percentage of NLPR3, ASC, and caspase-1 positive cells in the skin tissue of HFD animals was markedly increased (Figures 5(c)–5(h)). Also, we confirmed this conclusion in HFDs *in vitro* and found that p16 overexpression activated NLRP3 inflammasome pathway (Figures 5(i)–5(l)). Collectively, an excessive inflammatory response was induced in HFD animals, and was putatively linked with NLRP3 inflammasome signaling activation which promoted inflammatory infiltration and aggravated inflammatory responses.

### 3.6. p16 Knockout Ameliorates Integrin-Inflammasome Pathway Activation Induced by HFD

**Figure 6:**
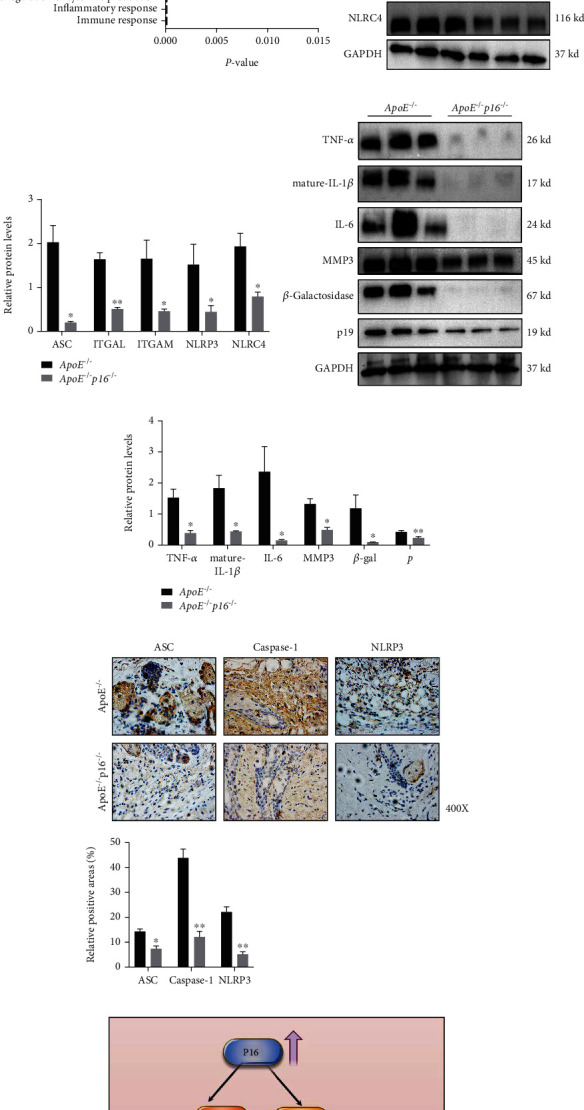
p16 knockout ameliorated activation of integrin-inflammasome pathway induced by high fat diet. (a, b) Further functional analysis of all upregulation differentially expressed genes from ApoE^−/−^ and ApoE^−/−^p16^−/−^ was performed with GO analysis and significantly enriched categories are highlighted; (c, d) expression levels and statistical figure of ASC, ITGAL, ITGAM, NLRP3, and NLRC4 in skin tissues from 20-week-old ApoE^−/−^ and ApoE^−/−^p16^−/−^ mice induced by HFD by western blotting (*n* = 3); (e, f) expression levels and statistical figures of TNF-*α*, mature-IL-1*β*, IL-6, MMP3, *β*-Galactosidase, and p19 in skin tissues from 20-week-old ApoE^−/−^ and ApoE^−/−^p16^−/−^ mice induced by HFD by western blotting (*n* = 3); (g, h) expression levels and statistical analysis of ASC, Caspase-1, and NLRP3 in skin tissues from 20-week-old ApoE^−/−^ and ApoE^−/−^p16^−/−^ mice induced by HFD by immunohistochemical staining (*n* = 3); (i) graphic abstract shown that p16 accumulation induced by HFD promoted activation of integrin-inflammasome pathway, thus aggravating accumulation of senescent cells and inflammaging in skin; values are mean ± SEM, ^∗^*p* < 0.05, ^∗∗^*p* < 0.01 compared with ApoE^−/−^ mice.

To investigate the mechanism of how p16 activates the inflammasome pathway, we analyzed all downregulated genes after p16 deletion by GO analysis in the DAVID website. This showed that p16 deletion inhibited genes associated with the positive regulation of integrin pathways (Figures 6(a) and 6(b)). Previous studies reported that integrin activation significantly activated inflammatory bodies and the maturation and release of IL-1*β* and IL-18 downstream molecules, generating an inflammatory microenvironment. From this, we hypothesized that p16 ablation could activate NLRP3 inflammasome by increasing ITGAM and ITGAL expression. Our western blotting data showed that NLRP3, NLRC4, ASC, ITGAL, and ITGAM expression levels were decreased after p16 knockout (Figures 6(c) and 6(d)). Also, we confirmed this conclusion via qPCR and found that inhibitory effect in inflammasome associated genes (NAIP5, NLRC4, TXK, and NAIP6) and genes related to integrin pathway (ITGB2, ITGAM, ITGB2L, and ITGAL) after p16 deficiency (Figure S4(a)). Additionally, when compared with ApoE^−/−^ fed with HFD diet, p16 deletion significantly inhibited the proinflammatory cytokine secretion of IL-1*β*, IL-6, TNF-*α*, and MMP3 in the skin (Figures 6(e) and 6(f)). We then detected the expression levels of SASP related genes (CCR3, CXCR5, and IL-1*β*) and found that p16 knockout decreased expression levels of these genes (Figure S4(b)). Also, p16 deletion decreased *β*-galactosidase and p19 expression levels (Figures 6(e) and 6(f)). Then, we detected expression levels of NLRP3, ASC, and Caspase-1, and found that p16 knockout decreased expression levels of NLRP3, ASC, and Caspase-1 (Figures 6(g) and 6(h)). Therefore, p16 deficiency inhibited integrin-inflammasome signaling and alleviated the inflammaging phenotype induced by HFD.

To confirm these conclusions *in vitro*, we cultured HDF cells and induced steatosis using sodium palmitate (10 mmol/L) and sodium oleate (10 mmol/L) for 24 h, and detected expression levels of ITGAM, ITGAL, and NLRC4 in cells. We found that p16 increased levels of ITGAM, ITGAL, and NLRC4 in HDF cells *in vitro* (Figures S4(c) and S4(d)). These results indicated that p16 overexpression could activate integrin-inflammasome pathways in skin.

To further confirm this conclusion, we detected *β*-galactosidase and p19 expression levels in skin via IHC staining, and the results showed that p16 KO significantly inhibited expression of *β*-galactosidase and p19 (Figures S5(a) and S5(b)). Additionally, we found that p16 knockout significantly decreased expression levels of ITGAM and ITGAL in skin (Figures S5(c) and S5(d)), also the expression levels of IL-1*β*, IL-6, and TNF-*α* were also decreased when p16 was deleted in ApoE^−/−^ mice (Figures S5(e) and S5(f)).

To further clarify if NLRP3 and NLRC4 inflammasome pathway activation was dependent on ITGAL and ITGAM, we designed and synthesized small interfering RNA (siRNA) molecules to knockdown mRNA and protein levels of ITGAL and ITGAM. We first detected mRNA and protein levels of ITGAL and ITGAM, and found that transfection with ITGAL or ITGAM siRNAs significantly inhibited their mRNA and protein expression levels in HDFs. Then, we examined the expression levels of inflammasome activation associated proteins (NLRP3, NLRC4, ASC, and caspase-1), and also the expression levels of IL-1*β*, IL-6, and TNF-*α* were measured using western blotting. Our results showed that when compared with NC groups, ITGAL or ITGAM knockdown inhibited the expression levels of NLRP3, NLRC4, ASC, and caspase-1 (Figures S6(a) and S6(b)). Additionally, IL-1*β*, IL-6, and TNF-*α* expression levels were decreased (Figure S6(b)). These data indicated that ITGAL and ITGAM were critical upstream regulators in activating the inflammasome pathway.

Overall, our results revealed that p16 overexpression activated integrin-inflammasome pathway and NF-*κ*B signaling to aggravate skin inflammaging.

## 4. Discussion

Human aging mechanisms are incredibly complicated, with many factors contributing to the process. Cell senescence has important roles in aging and age-related diseases [29]. Several key senescence-related proteins (p53 and p16) are increased during cell senescence, which is controlled via several signal transduction pathways, of which RB and p53-controlled pathways are of particular importance [30, 31]. At the core of these pathways, the protein products of several tumor suppressor genes are involved, including p16, p19, p21, and p53. In our study, when compared with the control group, the percentage of skin cells expressing senescence-related proteins, p16, p19, and *β*-gal was significantly increased in the HFD group, and suggested HFD increased senescence-related protein expression in the skin of ApoE^−/−^ mice to promote skin aging.

We also reported the accumulation of senescent cells in skin via p16 overexpression in the derma of HFD-induced ApoE^−/−^ mice. HFD induced this overexpression, increased apoptosis, and activated the NLRP3 inflammasome and NF-*κ*B signaling pathway to induce proinflammatory cytokine secretion; thus, aggravating inflammaging. Importantly, p16 deletion alleviated this process by decreasing senescent cells and inhibiting inflammaging. Molecular analyses also showed that p16 inhibited integrin-inflammasome pathway activation (Figure 6(i)).

p16 is a key mediator of cell senescence and was increased in skin during aging via oxidative stress and SASP [32, 33]. Also, p16 controls stem cell (SC) self-renewal in several tissues, and its deregulation may lead to aging. The elimination of senescent cells using ABT737 suppressed skin aging [34]. Zhou et al. revealed that p16 could bind with occluding to damage intestinal epithelial barrier in premature senescent mice model. p16 accumulation inhibited proliferation and hampered repair of intestinal epithelium, inducing increasing levels of proinflammatory cytokines and intestinal macrophage infiltration, finally disrupted microbial homeostasis [35]. However, the mechanism underlying the function of p16 in HFD-induced skin was unclear. To this end, we observed that p16 deletion alleviated senescence and fibrosis caused by HFD, by reducing integrin-inflammasome pathway activation.

Previous studies reported that the dermis of ApoE^−/−^ mice fed with HFD exhibited diffuse extracellular cholesterol crystallization and severe inflammatory cell infiltration. It was theorized that these free cholesterol crystals were sensed by macrophages and other innate immune skin cells to initiate a chronic low-grade inflammatory response [28, 36]. Fittingly, it was shown that cholesterol crystals acted as DAMP, leading to NLRP3 inflammasome activation [26, 37]. A recent study reported that chronic inflammasome activation and inflammatory senescence strongly contributed to the development and progression of age-related diseases [27, 38]. The NLRP3 inflammasome contains NLRP3 which interacts with its adapter, an apoptosis-associated speck-like protein containing a caspase-recruitment domain (ASC), to recruit and activate caspase-1, which then processes pro-IL-1*β* to mature IL-1*β* to accelerate inflammaging [39, 40]. To investigate this pathway in skin aging in response to HFD, we measured NLRP3, ASC, and caspase-1 inflammasome expression levels and found that all protein expression was markedly increased in the skin of ApoE^−/−^ mice fed with HFD, whereas p16 knockout rescued this process. Therefore, p16 induced NLRP3 signaling activation and caused skin aging in ApoE^−/−^ mice.

NLRC4 was originally described as a proapoptotic protein, capable of activating caspase-1 [41]. NLRC4 associates with procaspase-1 via CARD-CARD interactions, thereby triggering caspase-1 activation and inducing IL-1*β* and IL-18 secretion. Although NLRC4 has critical roles in host defenses, hyperactivated NLRC4 is potentially deleterious and may cause autoinflammatory disease [42]. Overexpressed NLRC4 in mice causes severe dermatitis, arthritis, and splenomegaly along with augmented neutrophil infiltration. Previous studies have also shown that NLRC4 is involved in tumor progression, including breast cancer and colitis-associated cancer [42, 43]. Ma et al. reported a critical role for the LCN2–SREBP2–NLRC4 axis in psoriasis pathogenesis [44]. But the role of NLRC4 in skin aging remains poorly characterized. In this study, we first reported that a HFD induced the aberrant activation of the NLRC4 inflammasome pathway to aggravate inflaming in skin. p16 may be a critical upstream activator of NLRC4. When transfection of ITGAL or ITGAM siRNA to knockdown expression levels of ITGAL and ITGAM in HDFs, we found that knockdown ITGAL and ITGAM in HDFs aborted activation of NLRP3 or NLRC4 inflammasome induced by p16 overexpression. This indicated that ITGAL and ITGAM may be main intermediate mediators of NLRC4 expression as regulated by p16.

Recent studies indicated that NF-*κ*B signaling, which is a major regulator of innate immunity, has crucial roles in SASP development and in inflammatory responses related to cell senescence [45–47]. It was also shown that p65 was phosphorylated at Ser536, and this transactivating modification was correlated with the increased expression and secretion of inflammatory markers [48]. In our study, the HFD significantly increased p65 phosphorylation at Ser536, suggesting the HFD may have promoted the classical inflammatory senescence-related activation of NF-*κ*B signaling, thereby contributing to skin aging.

Furthermore, p16 deletion attenuated inflammatory infiltration, inhibited inflammatory factor secretion, and inhibited integrin proinflammatory pathway activation. Additionally, after the HFD, p16 deletion inhibited apoptosis and effectively prevented further damage and fibrosis to the derma. Combined, p16 appears to have an important role preventing the progression of HFD-induced skin aging.

While we identified a key regulatory role and potential mechanism for p16 in regulating HFD-induced skin inflammaging, we used ApoE KO mice to construct a HFD-induced skin aging model and simulate pathological processes; however, this is a study weakness. Zhang et al. showed that ApoE KO mice suffered with spontaneous cutaneous xanthomatosis, and accelerated skin aging and frailty when fed a HFD. Additionally, mice showed spontaneous hyperlipidemia, which accelerated skin aging. Also, dyslipidemia can promote p16 protein expression [49]. Therefore, our research model may not fully reflect local p16 functions in the skin. In the future, the conditional KO of p16 and using physiological mice fed HFDs will be required to verify our conclusions. Furthermore, we showed p16 overexpression in skin after ApoE KO and p16 overexpression elevated expression of ApoE in fibroblast cells, which suggests the complex relationship between p16 and ApoE requires investigation.

## 5. Conclusion

In conclusion, we showed an HFD induced p16 overexpression and inflammaging in skin. p16 activated the NLRC4 and NLRP3 inflammasome pathway by increasing ITGAL and ITGAM expression. The p16 KO in skin rescued these pathological processes. Therefore, p16 could become a new target for the clinical treatment of skin aging when induced by HFD.

## Data Availability

The data used to support the findings of this study are available from the corresponding author JinDe Lin (hzljd@sohu.com) and Xin Gu (guxinjdfy@sohu.com) upon reasonable request.

## Abbreviations

p16: p16^INK4a^
*p16*-KO: *p16* homozygous
SASP: Senescence-associated secretory phenotype
HDFs: Human dermal fibroblasts
DAMPs: Damage-associated molecular patterns
SA-*β*-gal: Senescence associated-*β* galactosidase.

## Supplementary Materials

Supplementary 1 SI1: Figure S1: high fat diet causes aging and fibrosis in skin. (a) Statistical figure of *α*-SMA, Collagen I, and Collagen III in skin tissues from 20-week-old ApoE^−/−^ mice induced by ND and HFD by immunohistochemical staining (*n* = 3) to (Figures 1(g)–1(i)); (b, c) expression levels and statistical analysis of p19 and *β*-gal in skin tissues from 20-week-old ApoE^−/−^ mice induced by ND and HFD by immunohistochemical staining (*n* = 3); (d) expression levels and statistical figure of p21 and p19 in skin tissues from 20-week-old ApoE^−/−^ mice induced by ND and HFD by western blotting (*n* = 3); values are mean ± SEM, ^∗^*p* < 0.05; ^∗∗^*p* < 0.01; ^∗∗∗^*p* < 0.001 compared with ND diet mice.

Supplementary 2 Figure S2: high fat diet induced increased inflammation and SASP secretion. (a–c) Expression levels and statistical analysis of CD3, F4/80, and pp65 (S536) in skin tissues from 20-week-old ApoE^−/−^ mice induced by ND and HFD by immunohistochemical staining (*n* = 3). Values are mean ± SEM, ^∗^*p* < 0.05; ^∗∗^*p* < 0.01; ^∗∗∗^*p* < 0.001 compared with ND diet mice.

Supplementary 3 Figure S3: p16 increased expression level of ApoE in HDFs after inducing steatosis. (a, b) Expression levels and statistical analysis of ApoE in HDF cells, which were inducing steatosis for 24 h under medium containing sodium palmitate (10 mmol/L) and sodium oleate (10 mmol/L) and transfected with NC and p16 overexpression adenovirus were detected by western blotting (*n* = 3), ^∗^*p* < 0.05; ^∗∗^*p* < 0.01; ^∗∗∗^*p* < 0.001 compared with NC group.

Supplementary 4 Figure S4: p16 knockout alleviated activation of integrin-inflammasome pathway. (a, b) Relative mRNA expression levels of NAIP5, NLRC4, TXK, NAIP6, ITGB2, ITGAM, ITGB2L, ITGAL, CCR3, CXCR5, and IL-1*β* in skin tissues from 20-week-old ApoE^−/−^ and ApoE^−/−^p16^−/−^ mice induced by HFD were detected by Quantitative real-time polymerase chain reaction (qRT-PCR); (c, d) expression levels and statistical analysis of ITGAL, ITGAM, and NLRC4 in HDF cells, which were inducing steatosis for 24 h under medium containing sodium palmitate (10 mmol/L) and sodium oleate (10 mmol/L) and transfected with NC and p16 overexpression adenovirus, were detected by western blotting (*n* = 3), ^∗^*p* < 0.05; ^∗∗^*p* < 0.01; ^∗∗∗^*p* < 0.001 compared with NC group. Values are mean ± SEM, ^∗^*p* < 0.05; ^∗∗^*p* < 0.01; ^∗∗∗^*p* < 0.001 compared with ApoE^−/−^ diet mice.

Supplementary 5 Figure S5: p16 knockout alleviated activation of integrin-inflammasome pathway and inflammaging induced by high fat diet. (a, b) Relative levels of *β*-gal and p19 in skin tissues from 20-week-old ApoE^−/−^ and ApoE^−/−^p16^−/−^ mice induced by HFD were detected by immunohistochemical staining (*n* = 3); (c, d) relative levels of ITGAM and ITGAL in skin tissues from 20-week-old ApoE^−/−^ and ApoE^−/−^p16^−/−^ mice induced by HFD were detected by immunohistochemical staining (*n* = 3); (e, f) relative levels of IL-1*β*, IL-6, and TNF-*α* in skin tissues from 20-week-old ApoE^−/−^ and ApoE^−/−^p16^−/−^ mice induced by HFD were detected by immunohistochemical staining (*n* = 3); values are mean ± SEM, ^∗^*p* < 0.05; ^∗∗^*p* < 0.01; ^∗∗∗^*p* < 0.001 compared with ApoE^−/−^ diet mice.

Supplementary 6 Figure S6: knockdown of ITGAL and ITGAM could rescue activation of NLRP3 and NLRC4 inflammasome pathway after p16-overexpression. (a) Expression levels and statistical analysis of TNF-*α*, IL-1*β*, IL-6, NLRP3, ASC, NLRC4, p19, *β*-gal, and ITGAL in HDF cells, which were transfected with ITGAL small interfering RNA (si-ITGAL) for 48 h and inducing steatosis for 24 h under medium containing sodium palmitate (10 mmol/L) and sodium oleate (10 mmol/L), were detected by western blotting (*n* = 3), ^∗^*p* < 0.05; ^∗∗^*p* < 0.01; ^∗∗∗^*p* < 0.001 compared with NC + siNC group; ^&^*p* < 0.05; ^&&^*p* < 0.01; ^&&&^*p* < 0.001 compared with p16-OE + siNC group; (b) expression levels and statistical analysis of TNF-*α*, IL-1*β*, IL-6, NLRP3, ASC, NLRC4, p19, *β*-gal, and ITGAM in HDF cells, which were transfected with ITGAM small interfering RNA (si-ITGAM) for 48 h and inducing steatosis for 24 h under medium containing sodium palmitate (10 mmol/L) and sodium oleate (10 mmol/L), were detected by western blotting (*n* = 3); (c, b) expression levels and statistical analysis of TNF-*α*, IL-1*β*, IL-6, NLRP3, ASC, NLRC4, p19, *β*-gal, and ITGAM in skin tissue, which were treated with ITAGL or ITGAM antibody, were detected by western blotting (*n* = 3), ^∗^*p* < 0.05; ^∗∗^*p* < 0.01; ^∗∗∗^*p* < 0.001 compared with NC + siNC group; ^&^*p* < 0.05; ^&&^*p* < 0.01; ^&&&^*p* < 0.001 compared with p16-OE + siNC group.

Supplementary 7 SI2: Table S1: primer used for qPCR.

Supplementary 8 Table S2: chow composition of high fat diet and normal diet.

Supplementary 9 Table S3: composition of the saline solution reported in skin processing.

Supplementary 10 Table S4: sequence of small interfering RNA (siRNA) for ITGAL or ITGAM.
