# Supplementary figures and images for: p16INK4a Plays Critical Role in Exacerbating Inflammaging in High Fat Diet Induced Skin
Source: Oxid Med Cell Longev. 2022 Nov 21;2022:3415528. doi: 10.1155/2022/3415528 (PMC9706253; doi:10.1155/2022/3415528)

Figure S1 High fat diet causes aging and fibrosis in skin

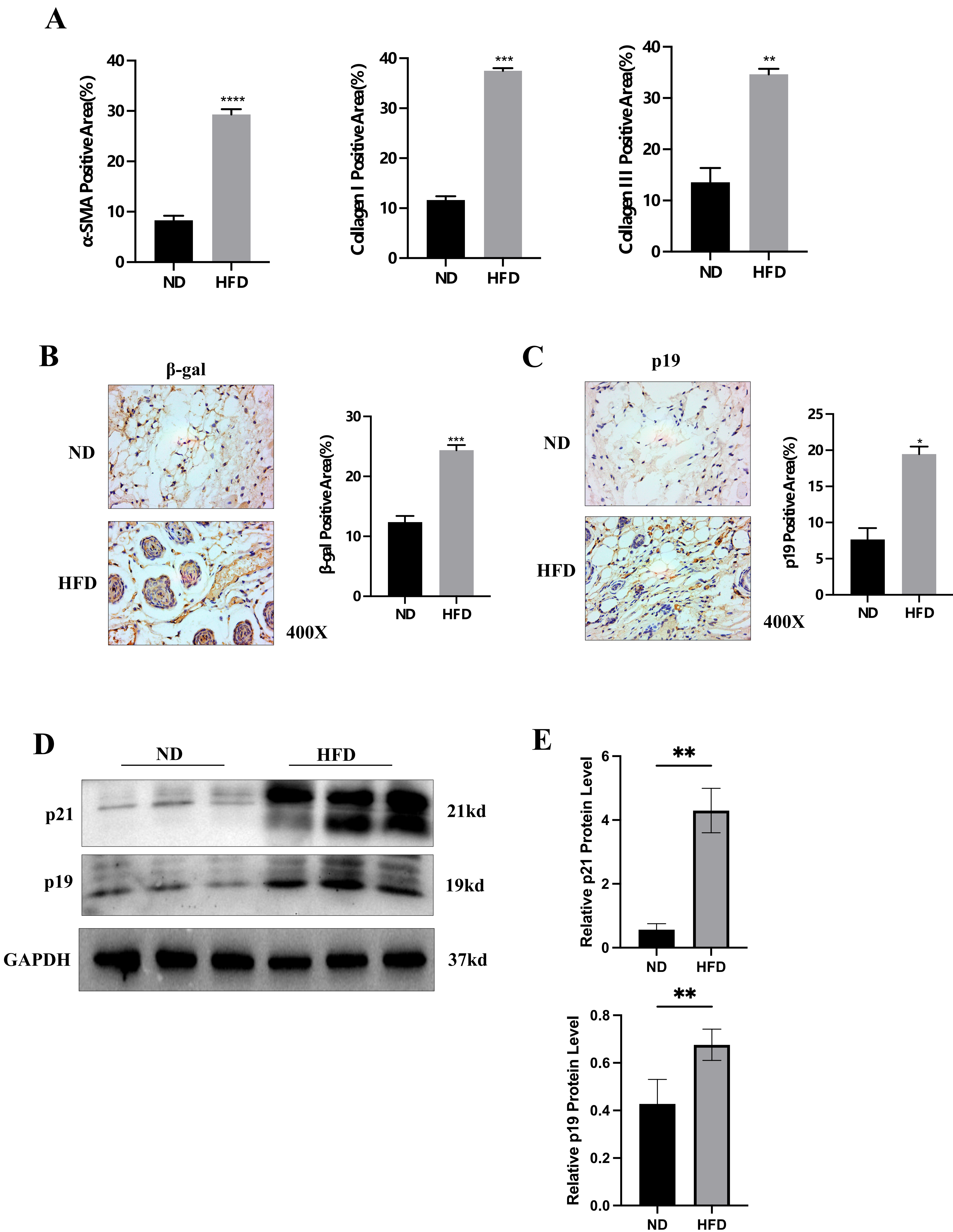

Supplement: Supplementary 1 — SI1: Figure S1: high fat diet causes aging and fibrosis in skin. (a) Statistical figure of α-SMA, Collagen I, and Collagen III in skin tissues from 20-week-old ApoE−/− mice induced by ND and HFD by immunohistochemical staining (n = 3) to (Figures 1(g)–1(i)); (b, c) expression levels and statistical analysis of p19 and β-gal in skin tissues from 20-week-old ApoE−/− mice induced by ND and HFD by immunohistochemical staining (n = 3); (d) expression levels and statistical figure of p21 and p19 in skin tissues from 20-week-old ApoE−/− mice induced by ND and HFD by western blotting (n = 3); values are mean ± SEM, ∗p < 0.05; ∗∗p < 0.01; ∗∗∗p < 0.001 compared with ND diet mice. [file 3415528.f1.pdf]

**Figure S2 High fat diet induced increased inflammation and SASP secretion**

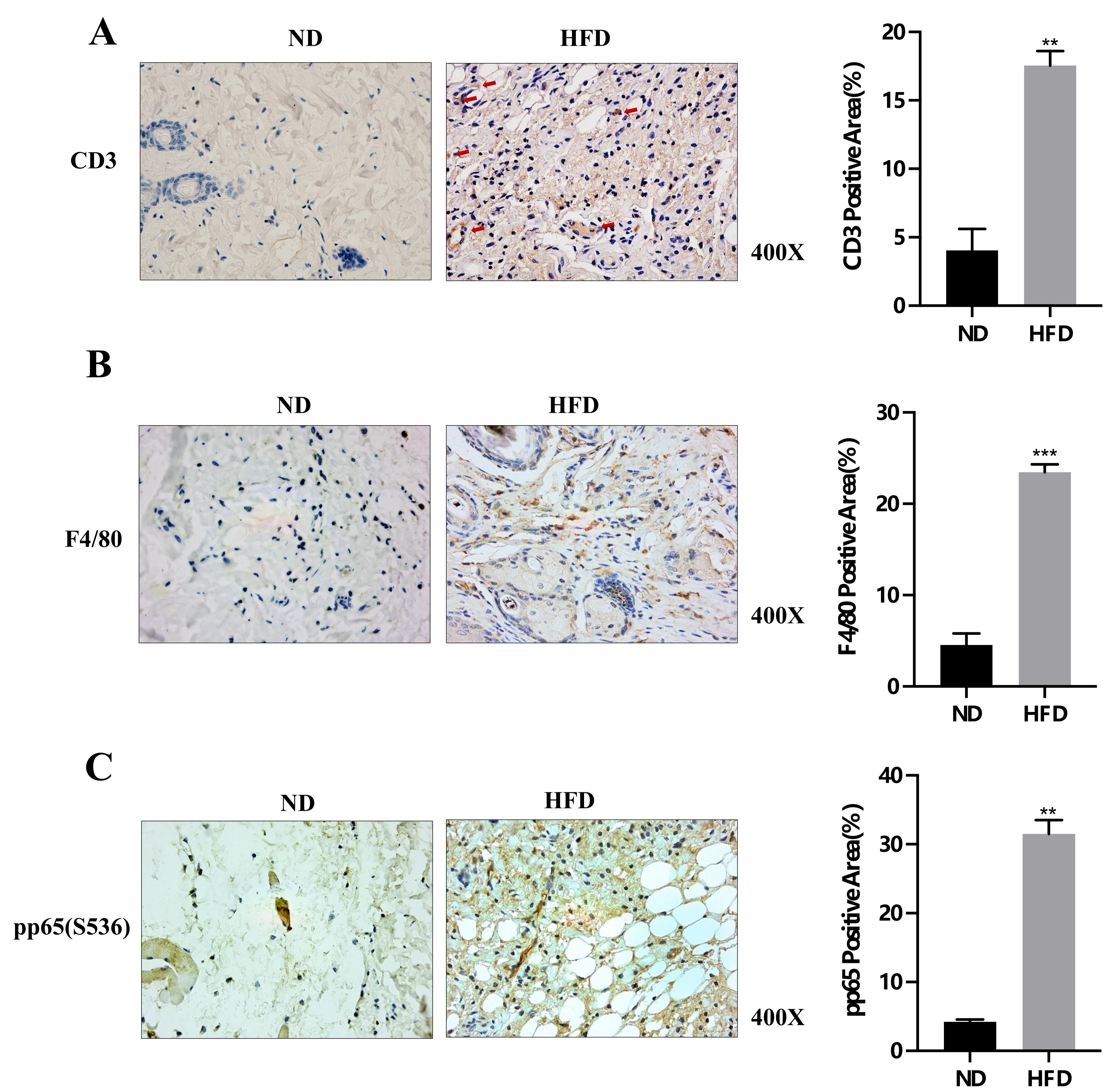

Supplement: Supplementary 2 — Figure S2: high fat diet induced increased inflammation and SASP secretion. (a–c) Expression levels and statistical analysis of CD3, F4/80, and pp65 (S536) in skin tissues from 20-week-old ApoE−/− mice induced by ND and HFD by immunohistochemical staining (n = 3). Values are mean ± SEM, ∗p < 0.05; ∗∗p < 0.01; ∗∗∗p < 0.001 compared with ND diet mice. [file 3415528.f2.pdf]

**Figure S3   p16 increased expression level of ApoE in HDFs after inducing steatosis**

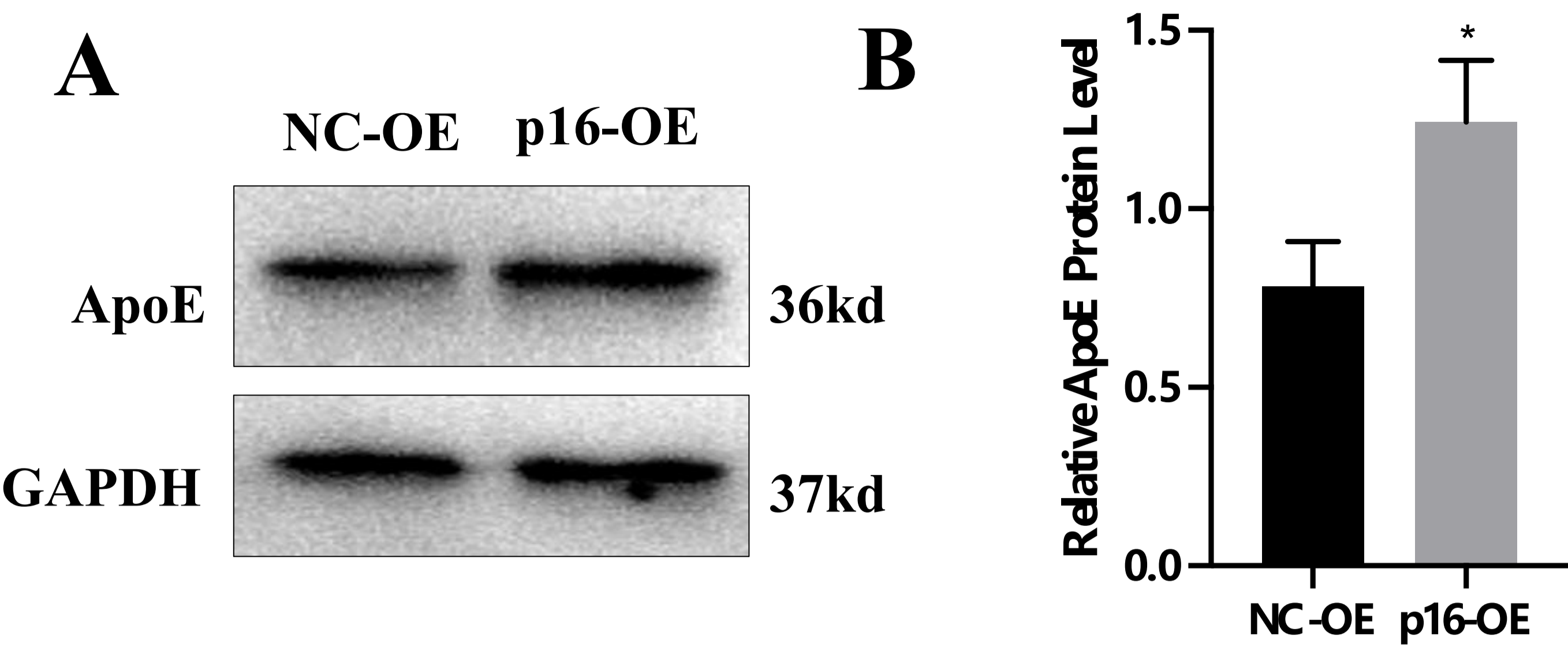

Supplement: Supplementary 3 — Figure S3: p16 increased expression level of ApoE in HDFs after inducing steatosis. (a, b) Expression levels and statistical analysis of ApoE in HDF cells, which were inducing steatosis for 24 h under medium containing sodium palmitate (10 mmol/L) and sodium oleate (10 mmol/L) and transfected with NC and p16 overexpression adenovirus were detected by western blotting (n = 3), ∗p < 0.05; ∗∗p < 0.01; ∗∗∗p < 0.001 compared with NC group. [file 3415528.f3.pdf]

Figure S4 p16 knockout alleviated activation of intergrin-inflammasome pathway

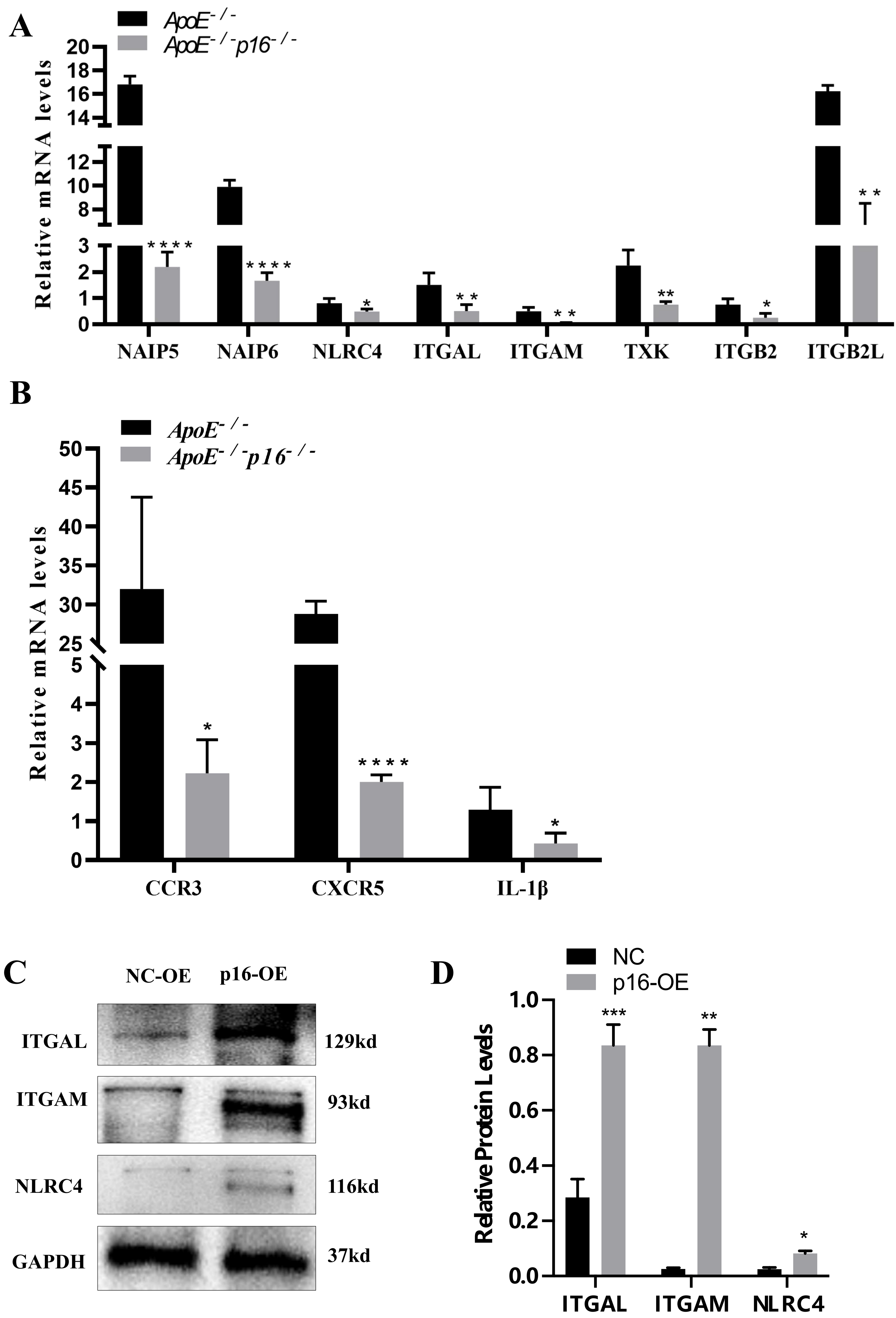

Supplement: Supplementary 4 — Figure S4: p16 knockout alleviated activation of integrin-inflammasome pathway. (a, b) Relative mRNA expression levels of NAIP5, NLRC4, TXK, NAIP6, ITGB2, ITGAM, ITGB2L, ITGAL, CCR3, CXCR5, and IL-1β in skin tissues from 20-week-old ApoE−/− and ApoE−/−p16−/− mice induced by HFD were detected by Quantitative real-time polymerase chain reaction (qRT-PCR); (c, d) expression levels and statistical analysis of ITGAL, ITGAM, and NLRC4 in HDF cells, which were inducing steatosis for 24 h under medium containing sodium palmitate (10 mmol/L) and sodium oleate (10 mmol/L) and transfected with NC and p16 overexpression adenovirus, were detected by western blotting (n = 3), ∗p < 0.05; ∗∗p < 0.01; ∗∗∗p < 0.001 compared with NC group. Values are mean ± SEM, ∗p < 0.05; ∗∗p < 0.01; ∗∗∗p < 0.001 compared with ApoE−/− diet mice. [file 3415528.f4.pdf]
