# Supplementary material for: p16INK4a Plays Critical Role in Exacerbating Inflammaging in High Fat Diet Induced Skin
Source: Oxid Med Cell Longev. 2022 Nov 21;2022:3415528. doi: 10.1155/2022/3415528 (PMC9706253; doi:10.1155/2022/3415528)

Figure S5 p16 knockout alleviated activation of intergrin-inflammasome pathway and inflammaging induced by high fat diet

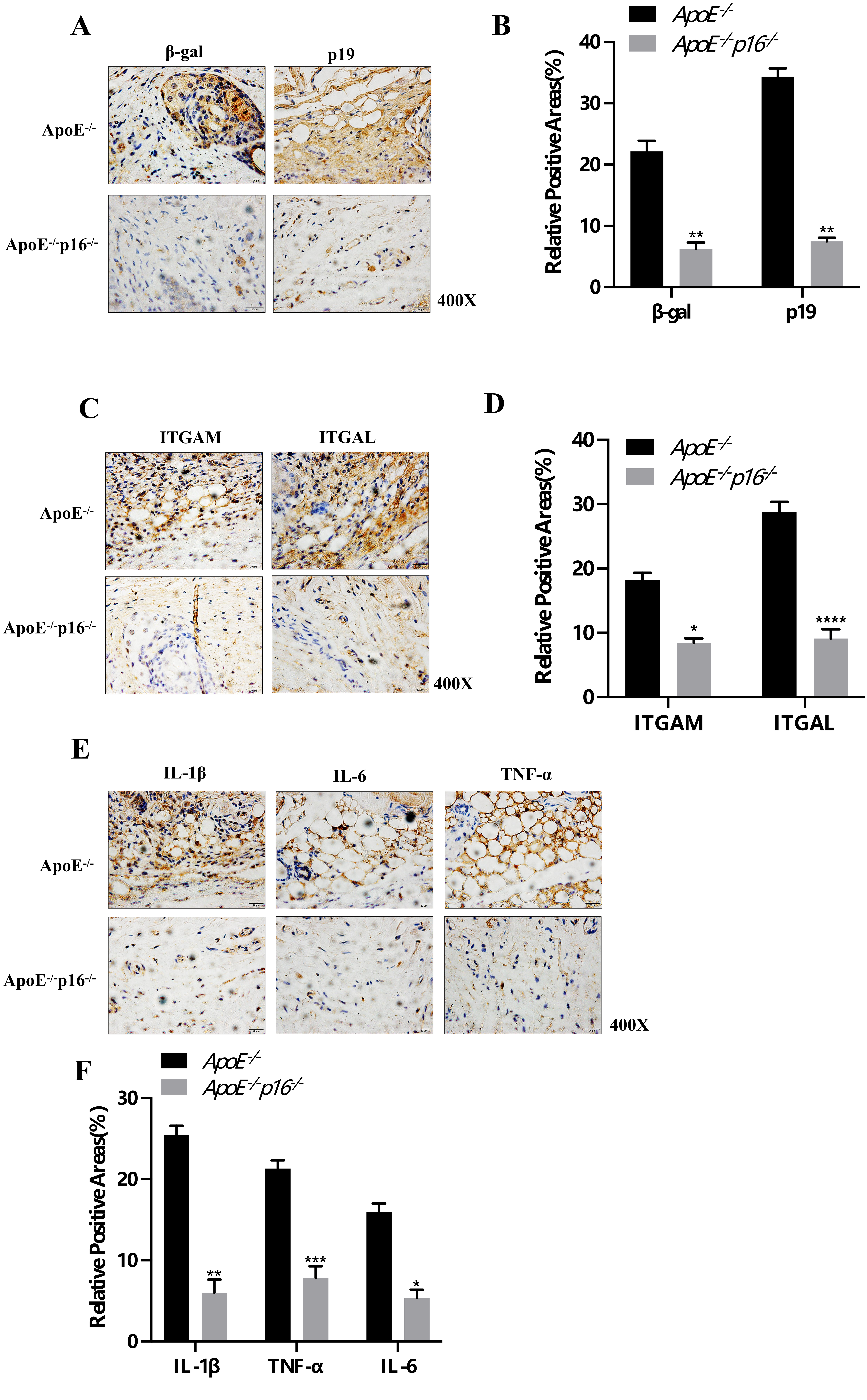

Supplement: Supplementary 5 — Figure S5: p16 knockout alleviated activation of integrin-inflammasome pathway and inflammaging induced by high fat diet. (a, b) Relative levels of β-gal and p19 in skin tissues from 20-week-old ApoE−/− and ApoE−/−p16−/− mice induced by HFD were detected by immunohistochemical staining (n = 3); (c, d) relative levels of ITGAM and ITGAL in skin tissues from 20-week-old ApoE−/− and ApoE−/−p16−/− mice induced by HFD were detected by immunohistochemical staining (n = 3); (e, f) relative levels of IL-1β, IL-6, and TNF-α in skin tissues from 20-week-old ApoE−/− and ApoE−/−p16−/− mice induced by HFD were detected by immunohistochemical staining (n = 3); values are mean ± SEM, ∗p < 0.05; ∗∗p < 0.01; ∗∗∗p < 0.001 compared with ApoE−/− diet mice. [file 3415528.f5.pdf]
