# Supplementary material for: p16INK4a Plays Critical Role in Exacerbating Inflammaging in High Fat Diet Induced Skin
Source: Oxid Med Cell Longev. 2022 Nov 21;2022:3415528. doi: 10.1155/2022/3415528 (PMC9706253; doi:10.1155/2022/3415528)

Figure S6 Knockdown of ITGAL and ITGAM could rescued activation of NLRP3 and NLRC4 inflammasome pathway after p16-overexpression

A

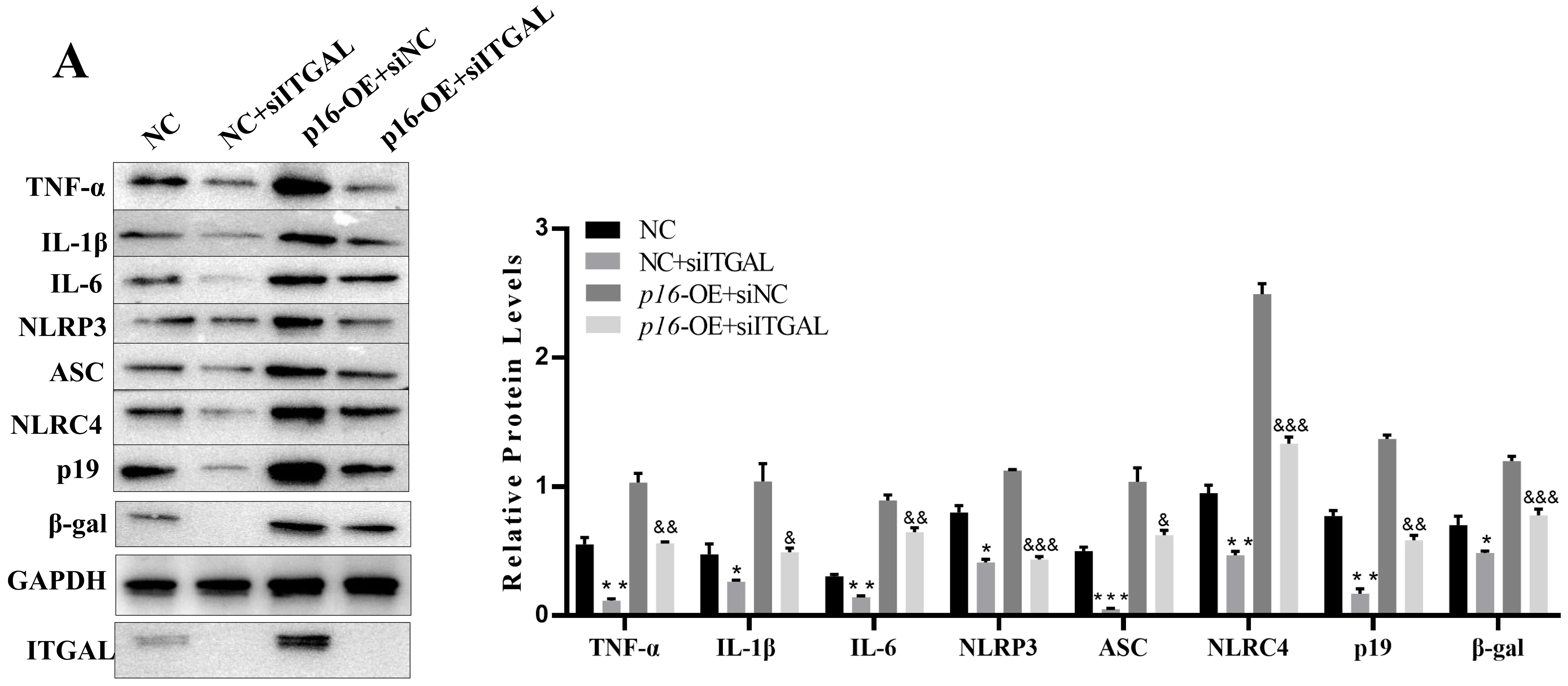

B

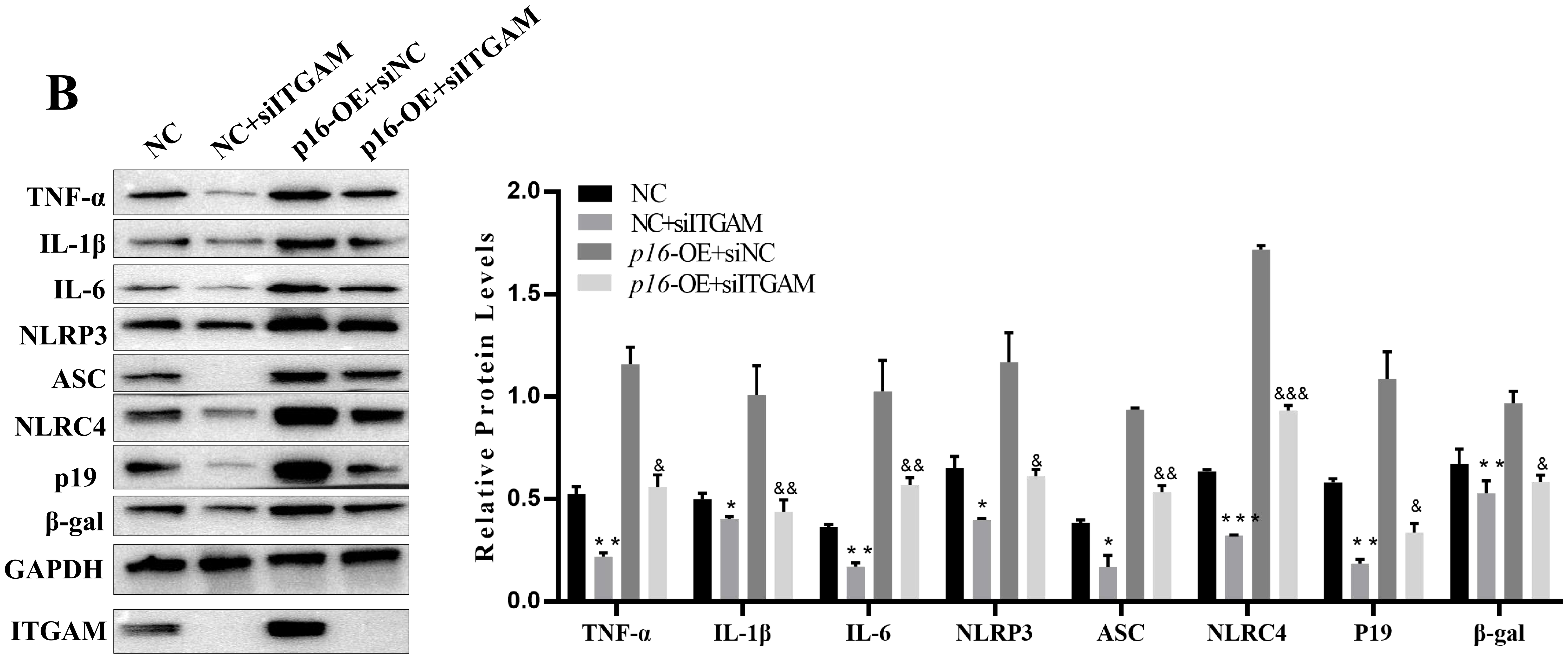

C

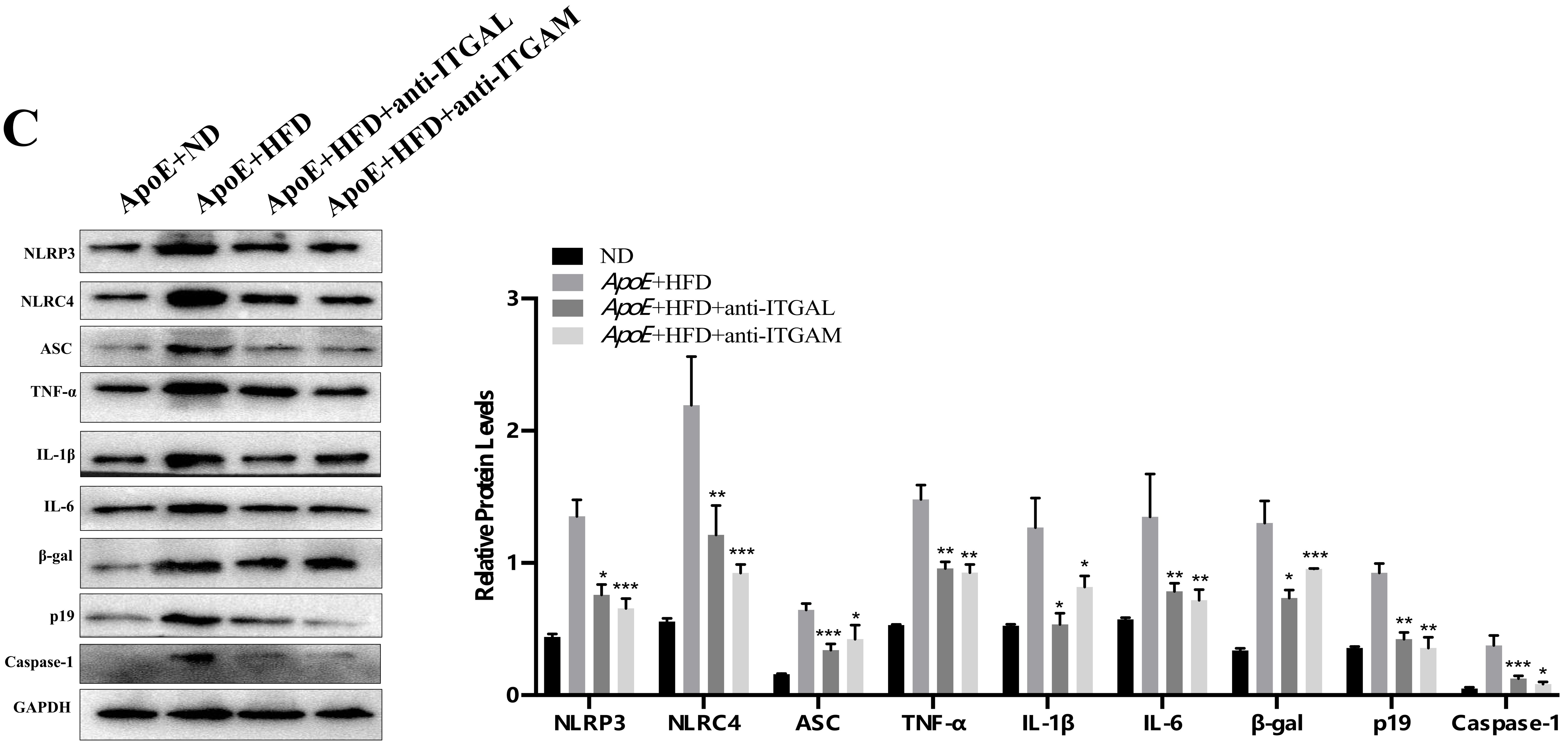

Supplement: Supplementary 6 — Figure S6: knockdown of ITGAL and ITGAM could rescue activation of NLRP3 and NLRC4 inflammasome pathway after p16-overexpression. (a) Expression levels and statistical analysis of TNF-α, IL-1β, IL-6, NLRP3, ASC, NLRC4, p19, β-gal, and ITGAL in HDF cells, which were transfected with ITGAL small interfering RNA (si-ITGAL) for 48 h and inducing steatosis for 24 h under medium containing sodium palmitate (10 mmol/L) and sodium oleate (10 mmol/L), were detected by western blotting (n = 3), ∗p < 0.05; ∗∗p < 0.01; ∗∗∗p < 0.001 compared with NC + siNC group; &p < 0.05; &&p < 0.01; &&&p < 0.001 compared with p16-OE + siNC group; (b) expression levels and statistical analysis of TNF-α, IL-1β, IL-6, NLRP3, ASC, NLRC4, p19, β-gal, and ITGAM in HDF cells, which were transfected with ITGAM small interfering RNA (si-ITGAM) for 48 h and inducing steatosis for 24 h under medium containing sodium palmitate (10 mmol/L) and sodium oleate (10 mmol/L), were detected by western blotting (n = 3); (c, b) expression levels and statistical analysis of TNF-α, IL-1β, IL-6, NLRP3, ASC, NLRC4, p19, β-gal, and ITGAM in skin tissue, which were treated with ITAGL or ITGAM antibody, were detected by western blotting (n = 3), ∗p < 0.05; ∗∗p < 0.01; ∗∗∗p < 0.001 compared with NC + siNC group; &p < 0.05; &&p < 0.01; &&&p < 0.001 compared with p16-OE + siNC group. [file 3415528.f6.pdf]
