# Supplementary material for: p16INK4a Plays Critical Role in Exacerbating Inflammaging in High Fat Diet Induced Skin
Source: Oxid Med Cell Longev. 2022 Nov 21;2022:3415528. doi: 10.1155/2022/3415528 (PMC9706253; doi:10.1155/2022/3415528)
Supplement: Supplementary 7 — SI2: Table S1: primer used for qPCR. [file 3415528.f7.docx]

**Table S1** Primers for qPCR

| **Name** | **S/AS** | **Sequence** |
| --- | --- | --- |
| *CXCL5* | S  AS | 5′-TCCAGCTCGCCATTCATGC-3′  5′-TTGCGGCTATGACTGAGGAAG-3 |
| *ITGAM* | S  AS | 5′-ATGGACGCTGATGGCAATACC-3′  5′-TCCCCATTCACGTCTCCCA-3′ |
| *ITGAL* | S  AS | 5′-CCAGACTTTTGCTACTGGGAC-3′  5′-GCTTGTTCGGCAGTGATAGAG-3′ |
| *ITGB2* | S  AS | 5′-CAGGAATGCACCAAGTACAAAGT-3′  5′-CCTGGTCCAGTGAAGTTCAGC-3′ |
| *ITGB2L* | S  AS | 5′-CACTGTCTCAGTTGTGTACCAAG-3′  5′-GCTCTGGTGTATCACAGCGAA-3′ |
| *IL-1β* | S  AS | 5′-GCAACTGTTCCTGAACTCAACT-3′  5′-ATCTTTTGGGGTCCGTCAACT-3′ |
| *NLRC4* | S  AS | 5′-ATCGTCATCACCGTGTGGAG-3′  5′-GCCAGACTCGCCTTCAATCA-3′ |
| *NAIP5* | S  AS | 5′-TGCCAAACCTACAAGAGCTGA-3′  5′-CAAGCGTTTAGACTGGGGATG-3′ |
| *NAIP6* | S  AS | 5′-TACAGGGAGTTTACAAGACCCC-3′  5′-AGTGGCCTGGAGAGACTCAG-3′ |
| *TXK* | S  AS | 5′-ACAGGTGAGAACTCAGATAAGCC-3′  5′-GGCAGGAAGTCATAAAGAGCCT-3′ |
| *GAPDH* | S  AS | 5’-CATTTCACTCAAGGTTGTCAGC-3’  5’-ATCATACTTGGCAGGTTTCTCC-3’ |

S, sense; AS, antisense
