# Supplementary material for: p16INK4a Plays Critical Role in Exacerbating Inflammaging in High Fat Diet Induced Skin
Source: Oxid Med Cell Longev. 2022 Nov 21;2022:3415528. doi: 10.1155/2022/3415528 (PMC9706253; doi:10.1155/2022/3415528)
Supplement: Supplementary 8 — Table S2: chow composition of high fat diet and normal diet. [file 3415528.f8.docx]

**Table S2** Chow composition of High fat diet and normal diet

| Normal diet | gm% | Kcal% |
| --- | --- | --- |
| Protein | 18.8 | 20.54 |
| Fat | 12.79 | 12.79 |
| Carbohydrate | 66.67 | 66.67 |

| High fat diet | gm% | Kcal% |
| --- | --- | --- |
| Protein | 26.2 | 20 |
| Fat | 34.9 | 60 |
| Carbohydrate | 26.3 | 20 |
