# Supplementary material for: p16INK4a Plays Critical Role in Exacerbating Inflammaging in High Fat Diet Induced Skin
Source: Oxid Med Cell Longev. 2022 Nov 21;2022:3415528. doi: 10.1155/2022/3415528 (PMC9706253; doi:10.1155/2022/3415528)
Supplement: Supplementary 9 — Table S3: composition of the saline solution reported in skin processing. [file 3415528.f9.docx]

**Table S3** Composition of the saline solution reported in skin processing

| Components | Molecular Weight | Concentration(mg/L) | mM |
| --- | --- | --- | --- |
| Inorganic Salts |  |  |  |
| Potassium Phosphate Monobasic(KH2PO4) | 136.0 | 144.0 | 1.0588236 |
| Sodium Chloride(NaCl) | 58.0 | 9000.0 | 155.17241 |
| Sodium Phosphate dibasic(Na2HPO4-7H2O) | 268.0 | 795.0 | 2.966418 |
