# Supplementary material for: p16INK4a Plays Critical Role in Exacerbating Inflammaging in High Fat Diet Induced Skin
Source: Oxid Med Cell Longev. 2022 Nov 21;2022:3415528. doi: 10.1155/2022/3415528 (PMC9706253; doi:10.1155/2022/3415528)
Supplement: Supplementary 10 — Table S4: sequence of small interfering RNA (siRNA) for ITGAL or ITGAM. [file 3415528.f10.docx]

**Table S4** Sequence of small interfering RNA(siRNA) for ITGAL or ITGAM

siRNAs against Human ITGAL

| Name | S/AS | Sequence |
| --- | --- | --- |
| siRNA 1  Negative control siRNA | S  AS  S  AS | 5′-AUACCUUUGGUGCCAUCAAUUdTdT-3′  5′-AAUUGAUGGCACCAAAGGUAUdTdT-3′  5′-UUCUCCGAACGUGUCACGUTT-3′  5′-ACGUGACACGUUCGGAGAATT-3′ |

siRNAs against Human ITGAM

| Name | S/AS | Sequence |
| --- | --- | --- |
| siRNA 1  Negative control siRNA | S  AS  S  AS | 5′-CAACUGUGAUGGAGCAAUUAAdTdT-3′  5′-UUAAUUGCUCCAUCACAGUUGdTdT-3′  5′-UUCUCCGAACGUGUCACGUTT-3′  5′-ACGUGACACGUUCGGAGAATT-3′ |
